# Identification and Expression Analysis of Transcription Factor Family in Highland Barley Seedlings Under Na_2_SeO_3_ Treatment

**DOI:** 10.3390/life16020255

**Published:** 2026-02-02

**Authors:** Xiaozhuo Wu, Feng Qiao, Guigong Geng, Jianxia Ma, Huichun Xie

**Affiliations:** 1 Key Laboratory of Tibetan Plateau Medicinal Plant and Animal Resources, School of Life Sciences, Qinghai Normal University, Xining 810008, China; xiaozhuo0623@163.com (X.W.); majianxia0926@163.com (J.M.); 2 Academy of Plateau Science and Sustainability, Qinghai Normal University, Xining 810008, China; 3 Qinghai South of Qilian Mountain Forest Ecosystem Observation and Research Station, Huzhu, Haidong 810500, China; 4 National Forestry Grassland Qinghai Tibet Plateau Characteristic Forest and Grassland Germplasm Resources Protection and Utilization Engineering Technology Research Center, Xining 810008, China; 5 Academy of Agricultural and Forestry Sciences, Qinghai University, Xining 810016, China; genggg-298@163.com

**Keywords:** highland barley, transcription factor, bioinformatics, expression analysis

## Abstract

Transcription factors (TFs) are ubiquitously distributed in plants and play pivotal roles in regulating plant growth and development. The present study aims to elucidate the function of transcription factors (TFs) in highland barley’s response to selenium stress. The results show that 89, 218, 141, 92, 23, and 34 genes were identified from the bHLH, MYB, NAC, WRKY, GATA, and HSF families, respectively. We analyzed the physicochemical properties of the transcription factor family, including amino acid number and molecular weight, theoretical PI, instability index, hydrophilicity index, and subcellular location. The majority of proteins encoded by these gene families are hydrophilic and predominantly localized in the nucleus. Structural analysis demonstrates that each family contains conserved motifs and domains. Most bHLH genes, such as *KAE8811666.1* and *KAE8789390.1*, contain bHLH_SF superfamily domains. 45 MYB genes possess the myb_SHAQKYF domain. Most NAC genes possess typical NAM domains. Most WRKY proteins showed the WRKY superfamily domain. The 22 members of GATA possess the ZnF_GATA domain. HSF gene family showed that 24 gene family members contained HSF domains. Systematic evolutionary analysis indicates that the bHLH and NAC families can each be divided into nine subfamilies, while the remaining four families are categorized into five to eight subfamilies, respectively. Based on transcriptome data, under low selenium treatment, 56.25%, 76%, 67.39%, 47.37%, 50%, and 56.25% of the genes belonging to the bHLH, MYB, NAC, WRKY, GATA, and HSF transcription factor families were significantly upregulated, respectively. In contrast, under high selenium treatment, the proportions of upregulated genes in these families were 81.25%, 80%, 65.22%, 63.16%, 75%, and 75%, respectively. Additionally, qRT-PCR results were consistent with the trends of the transcriptome expression data, corroborating the reliability and accuracy of the transcriptomic findings. These results elucidate the molecular characteristics and response patterns of six transcription factor families to selenium stress in highland barley, laying a foundation for further in-depth research on the functions of transcription factors in highland barley plants.

## 1. Introduction

Transcription factors (TFs) are a class of important regulatory factors found in eukaryotes that regulate the expression of specific genes by interacting with cis-acting elements. These factors help mediate the adaptation to environmental stress [1]. bHLH proteins constitute one of the most extensive transcription factor families in plants [2]. The bHLH transcription factors were first identified in maize [3]. Proteins belonging to the bHLH family usually possess a conserved bHLH domain of around 60 amino acids, which is split into two functionally distinct parts: the basic region and the helix-loop-helix (HLH) region [4]. The basic region, which is situated close to the N-terminus, typically comprises 10–15 amino acids and functions in the recognition of specific DNA sequences [5]. The C-terminal HLH region, spanning about 40 amino acids, is composed of two relatively conserved α-helices linked by a loop of variable length. This region is enriched in hydrophobic amino acids, which facilitates the formation of homodimers or heterodimers necessary for their biological activities [6]. Studies have confirmed that the conserved domain of this gene family has been highly conserved throughout plant evolution (dating back 400 million years), and the family size has gradually expanded during evolution [7]. MYB transcription factors constitute one of the most ubiquitous and functionally diverse gene families in plants, playing crucial roles in regulating growth, development, metabolic pathways, and stress tolerance [8]. Its core structure consists of a highly conserved MYB DNA-binding domain, which usually contains one to four imperfect repeat units, each approximately 50–53 amino acids in length. Each repeat folds into a helix-turn-helix (HTH) conformation, allowing MYB proteins to bind specifically to target DNA sequences and thereby modulate the expression of downstream genes [9]. The NAC and WRKY families are among the largest plant transcription factor families. A typical NAC protein contains a conserved NAC domain at the N-terminus for DNA binding and nuclear localization, as well as a variable C-terminal region with transcriptional regulatory activity [10]. As a complex plant-specific family, NAC genes are abundantly present in many species. A large number of NAC transcription factors have been identified in various plants, including *Asparagus officinalis* L. [11], *Hylocereus undatus* (Haw.) Britton & Rose [12], and *Zanthoxylum bungeanum Maxim*. [13]. WRKY proteins are a class of transcription factors characterized by a conserved WRKY domain, playing significant roles as transcriptional repressors and activators [14]. The WRKY domain consists of four β-strands and is composed of approximately 60 highly conserved amino acid residues [15]. Both families play important roles in plant growth and development as well as in responses to abiotic stress. The GATA family comprises a large set of transcription factors capable of recognizing the GATA motif and binding specifically to promoter sequences of the form (A/T)GATA(A/G) [16]. The majority of these proteins contain one or two strongly conserved type IV zinc finger motifs, characterized by the sequence CX_2_CX_17–20_CX_2_C [17,18]. In plants, the GATA gene *NTL1* was first isolated from tobacco [19]. With the exploration of genomic data, an increasing number of plant HSF gene families have been identified, such as in *T. aestivum* [20], *H. vulgare* [21], and *Z. mays* [22]. HSFs possess five essential functional domains: an N-terminal DNA-binding domain (DBD), an oligomerization domain (OD), a nuclear localization signal (NLS), a nuclear export signal (NES), and a C-terminal activation domain (CAD) [23,24]. HSFs play a key role in mediating plant responses to various abiotic stresses, regulating plant stress resistance through mechanisms such as phosphorylation, ubiquitination, and hormone signaling [25,26].

Previous studies have shown that the bHLH transcription factor family plays a significant regulatory role in heavy metal stress [27]. MYB, NAC, WRKY, and GATA family members can contribute to the modulation of multiple abiotic stress responses [28]. *ZmbHLH105* can enhance maize tolerance to manganese stress by mediating the expression of manganese/iron-related transporters and regulating the reactive oxygen species (ROS) scavenging through antioxidant mechanisms [29]. Sorghum *SbbHLH85* enhances salt tolerance [30]. In Scutellaria baicalensis, SbMYB3 directly activates SbFNSII-2 transcription, which in turn promotes the synthesis of flavonoid compounds specific to the root [31]. Increased expression of SlGATA17 improves drought tolerance in tomatoes by enhancing the activity of the phenylpropanoid biosynthesis pathway [32]. It has been reported that HSF expression is upregulated under abiotic stresses such as salinity and drought due to activation by reactive oxygen species (ROS), thereby increasing antioxidant enzyme activity to maintain ROS homeostasis within the plant [33,34]. The response to selenium stress is not only related to selenium metabolism and oxidative homeostasis but also involves a complex process of multiple signal transduction pathways and gene expression regulation, enabling adaptation to various abiotic stresses such as salt stress, temperature stress, and heavy metal stress [35]. In this process, after the stress signals are perceived and transduced, many transcription factors are rapidly induced to be expressed, reflecting the important role of transcription factors in both biotic and abiotic stresses [36,37,38]. However, few studies have focused on the role of transcription factors in response to selenium stress. Research by Wu et al. has shown that both the overexpression and mutation of the transcription factor *WRKY47* in Arabidopsis increased the plant’s sensitivity to selenium [39].

Highland barley (*Hordeum vulgare* L.) is a unique crop of the Tibetan Plateau, primarily distributed in alpine regions above 3000 m [40]. In 2019, the production of highland barley in its main producing areas, Tibet and Qinghai, was 792,900 tons and 444,100 tons, respectively, accounting for over 80% of the total highland barley production [41]. Highland barley exhibits strong cold tolerance, a short growth cycle, broad adaptability, early maturity, and high yield, making it well-suited for cultivation in the cool plateau climate [42]. Compared to other cereal crops, highland barley has better nutritional value due to its higher protein, vitamin, and fiber content, particularly β-glucan. β-glucan has various physiological effects, such as lowering blood sugar and fat levels, reducing cholesterol, preventing colon cancer, and enhancing immunity [43]. As an important crop in the Tibetan Plateau region, the cultivation industry of highland barley plays a significant role in the local agricultural economic development, and its unique resilience formed under harsh environmental conditions makes it a valuable material for studying the mechanisms of crop resistance to adversity.

Existing studies have confirmed that transcription factor families such as bHLH, MYB, NAC [44], and WRKY [45] play important roles in abiotic stress responses, but few studies have investigated the functions and regulatory mechanisms of these transcription factors in highland barley’s response to selenium stress. Highland barley, as a unique crop in the Qinghai–Tibet Plateau, has its unique adaptation mechanism to selenium stress. In our previous research, we explored the relationship between transcription factors and organic acid metabolites in green seedlings under selenium stress. We further screened 26 differentially expressed genes (DEGs) related to Na_2_SeO_3_ concentration. Based on correlation analysis, there were six genes in the bHLH family, five in MYB, three in NAC, five in WRKY, and three in the GATA and HSF families that showed positive correlations with 30 differential organic acid metabolites. However, there is a lack of systematic identification and expression analysis of the above six transcription factor families in highland barley under selenium stress. This research gap limits the in-depth understanding of the molecular mechanism of highland barley response to selenium stress. Therefore, in this study, we systematically analyzed the bHLH, MYB, NAC, WRKY, GATA, and HSF transcription factor gene families in highland barley seedlings using a bioinformatics platform. Our analysis covered multiple aspects, including the identification of gene family members, prediction of protein physicochemical properties, analysis of conserved motifs and gene structures, and functional characterization of cis-acting elements in promoter regions. By integrating transcriptome data from seedlings treated with different concentrations of Na_2_SeO_3_, we further investigated the expression patterns of these genes under selenium stress. These results lay a solid foundation for future studies on the selenium response mechanisms and biological functions of these transcription factor families in hulless highland barley, and also provide theoretical support for the breeding of stress-resistant and high-quality germplasm resources.

## 2. Materials and Methods

### 2.1. Identification of Transcription Factor Family Members

Based on the reference transcriptome data available in our research group, the hulless highland barley genome data was retrieved from GCA_004114815.1_Hulless_Barley_ass.V2_genomic.fna (https://www.ncbi.nlm.nih.gov/assembly/GCA_004114815.1, accessed on 3 July 2025). We obtained the corresponding hidden Markov model (HMM) files from the Pfam database [46] on the InterPro website, specifically for the following domains: bHLH (PF00010), MYB (PF00249), NAC (PF02365), WRKY (PF03106), HSF (PF00447), and GATA (PF00320). Using HMMER software(Version 3.2.1), we searched for proteins matching these domain models. Subsequently, we constructed protein databases for these six families in wheat and rice using BLAST software (https://blast.ncbi.nlm.nih.gov/, accessed on 5 July 2025). We aligned the protein sequences of hulless highland barley with those of the wheat and rice protein families to identify all potential protein sequences and obtain candidate proteins. We then took the intersection of the prediction results from HMMER and BLAST software [47]. Finally, after integrating the aforementioned results, we employed the NCBI CD-Search online tool (https://www.ncbi.nlm.nih.gov/Structure/cdd/wrpsb.cgi, accessed on 8 July 2025) for domain validation [48], which yielded the candidate family protein sequences.

### 2.2. Analysis of Physicochemical Properties of Transcription Factor Family Member Proteins

The physicochemical properties of the protein sequences from the six families in highland barley seedlings were analyzed using the online ExPASy ProtParam tool [49] (http://web.expasy.org/protparam/, accessed on 11 July 2025). The predicted parameters included the number of amino acids, relative molecular weight, theoretical isoelectric point (pI), instability index (II), aliphatic index (AI), and grand average of hydropathicity (GRAVY). Additionally, subcellular localization was predicted using the WoLF PSORT software (https://www.genscript.com/wolf-psort.html, accessed on 12 July 2025) [50].

### 2.3. Conserved Motifs, Conserved Domains, and Gene Structure Analysis of Transcription Factor Family Genes

In this study, we used the MEME tool (https://meme-suite.org/meme/index.html, accessed on 15 July 2025) to analyze conserved motifs, setting the number of motifs to 10 and keeping the remaining parameters at their default settings. Subsequently, we visualized these conservative motifs using the Gene Structure View program within TBtools (v1.082). To predict the core conserved domains of the members of the six gene families, we used the online HMMER platform and organized the results accordingly. Finally, we utilized the ‘Gene Structure View (advanced)’ plugin in TBtools software (v1.082) to visualize the identified conserved motifs and domains [51,52].

### 2.4. Analysis of Cis-Acting Elements of Transcription Factor Family Genes

Initially, the genome annotation files and genome sequences were loaded into the Gtf/Gff3 Sequences Extract module of TBtools. The Fasta Extract tool was used to retrieve the 2000 bp upstream regions of the target genes, which were then uploaded to the PlantCARE website (http://bioinformatics.psb.ugent.be/webtools/plantcare/html/, accessed on 5 September 2025) for cis-acting element prediction [53]. The resulting data were visualized and analyzed with Tbtools [52].

### 2.5. Construction of the Evolutionary Tree of Transcription Factor Gene Family System

To analyze the evolutionary relationships of the transcription factors, this study used the MUSCLE Wrapper module in TBtools software to perform multiple sequence alignment of the protein sequences from the six families of highland barley seedlings and wheat. Based on the alignment results, we used the IQ-TREE program [54] integrated in TBtools to construct a Maximum Likelihood (ML) phylogenetic tree. Finally, the phylogenetic tree was visualized and optimized using the Interactive Tree of Life (iTOL) online platform (https://itol.embl.de/, accessed on 11 September 2025), with color labeling to distinguish species-specific branches and conserved subgroups [55].

### 2.6. Expression Patterns of Transcription Factor Family Genes in Highland Barley

This study analyzed the gene expression patterns of the six highland barley transcription factor families using transcriptome data previously collected by our research group. Sodium selenite treatments at three different concentrations were used: CK (control group), T1 (0.02 g/kg), and T2 (0.2 g/kg) to assess the gene expression levels in highland barley samples exposed to different concentrations of sodium selenite. The concentrations of sodium selenite used in this study (T1: 0.02 g/kg, T2: 0.2 g/kg) were selected based on our research group’s previous physiological studies on barley seedlings, which supported the rationality of the selenium concentration settings [56]. The data on gene expression patterns were sourced from the published transcriptome data under NCBI BioProject accession number PRJNA1266111. The criteria for screening differentially expressed genes (DEGs) were |log2FoldChange| > 1 and a significant *p*-value < 0.05. The OmicShare tool was utilized to convert the gene expression data into visual heatmaps, facilitating the analysis of gene expression patterns.

### 2.7. qRT-PCR for Gene Expression Validation

To verify the reliability of the transcriptome data, quantitative reverse transcription PCR (qRT-PCR) was performed on an iQ5 Real-Time PCR Detection System (Bio-Rad, Hercules, CA, USA) using samples plated in a 96-well plate. Gene-specific primers were designed with Primer Premier 5 software and synthesized by BGI Corporation (Beijing, China), with EF1-α serving as the reference gene. Each qRT-PCR reaction mixture contained 6.8 μL of RNase-free water, 0.4 μL each of forward and reverse primers, 10 μL of 2× SYBR real-time PCR premix, 0.4 μL of ROX Reference Dye II, and 2 μL of diluted cDNA. The amplification program began with an initial denaturation at 95 °C for 5 min, followed by 40 cycles of 95 °C for 15 s and 60 °C for 30 s, and ended with a melting curve analysis. Relative expression levels were determined using the 2^−ΔΔCt^ method [57]. Three biological replicates were included in the qRT-PCR analysis. One-way ANOVA was performed to compare the control and Se-treated groups using IBM SPSS Statistics 27. Multiple comparisons were performed using the least significant difference (LSD) test for significant differences.

## 3. Results

### 3.1. Family Member Identification and Physicochemical Properties of Six Transcription Factors

Based on genomic and transcriptomic data from highland barley, we identified six transcription factor families: bHLH, MYB, NAC, WRKY, GATA, and HSF. Through Pfam and NCBI BLAST analyses, a total of 89 bHLH family members were identified and named bHLH1 to bHLH89. These proteins varied in length from 69 to 882 amino acids (aa), with molecular weights ranging from 8093.80 to 96,224.86 Da. Their theoretical isoelectric points ranged from 4.68 to 11.57. Among these proteins, two were classified as stable, whereas 87 were predicted to be unstable. Based on the aliphatic index and grand average of hydropathicity, 88 bHLH proteins were identified as hydrophilic, while one was classified as hydrophobic. Subcellular localization predictions indicated that most bHLH proteins are primarily localized in the nucleus, followed by chloroplasts and cytoplasm. A small number of proteins were also predicted to localize in the peroxisome, endoplasmic reticulum, and mitochondria (Table S1).

A total of 218 MYB gene family members were identified and designated as MYB1 to MYB218. The number of amino acids in these proteins ranged from 72 to 1088, with corresponding molecular weights varying from 7925.87 to 121,042.43 Da. Their theoretical isoelectric points spanned 4.67 to 10.74, and stability analysis predicted 12 proteins to be stable and 206 to be unstable. Based on the aliphatic index and hydrophilicity index, all 218 MYB proteins were classified as hydrophilic. Subcellular localization predictions indicated that most MYB proteins are primarily localized in the nucleus, followed by chloroplasts and the cytoplasm, with a small number found in peroxisomes and mitochondria (Table S2).

A total of 141 NAC gene family members were identified and named NAC1 through NAC141. Our analysis showed that these proteins contained between 92 and 863 amino acids, with corresponding molecular weights ranging from 10,521.89 to 96,248.97 Da. The theoretical isoelectric points varied from 4.29 to 11.69, and stability predictions classified 21 proteins as stable and 120 as unstable. Based on the grand average of hydropathicity (GRAVY) and aliphatic index, all 141 NAC proteins were determined to be hydrophilic. Subcellular localization predictions suggested that most NAC proteins were targeted to the nucleus, with smaller numbers localized in chloroplasts and the cytoplasm. A limited number of proteins were also predicted to reside in peroxisomes, the Golgi apparatus, and mitochondria (Table S3).

Ninety-two members of the WRKY gene family were selected and designated as WRKY1 to WRKY92. It was observed that the number of amino acids in these proteins varied from 103 to 1376. The molecular weights ranged from 11,126.69 to 155,767.22 Da. The theoretical isoelectric points varied between 5.1 and 10.37, with two proteins identified as stable and ninety as unstable. Based on the fat coefficient and hydrophilicity index, all 92 WRKY proteins are classified as hydrophilic. Predictions regarding subcellular localization indicated that WRKY proteins are primarily localized in the nucleus, followed by the chloroplasts and cytoplasm. A smaller number are localized in peroxisomes, the endoplasmic reticulum, mitochondria, and cell membranes (Table S4).

A total of 23 GATA gene family members were identified and designated as GATA1 to GATA23. These proteins contained between 136 and 818 amino acids, with molecular weights ranging from 15,101.47 to 92,854.14 Da. Their theoretical isoelectric points varied from 4.6 to 10.08, and all 23 proteins were predicted to be unstable. Based on the aliphatic index and hydrophilicity values, all GATA proteins were classified as hydrophilic. Subcellular localization predictions suggested that GATA proteins are primarily targeted to the nucleus, with additional localization in chloroplasts and mitochondria (Table S5).

A total of 34 heat shock factor (HSF) gene family members were identified and named HSF1 to HSF34. These proteins contained between 96 and 569 amino acids, with molecular weights ranging from 11,136.81 to 63,740.84 Da. The theoretical isoelectric points varied from 4.67 to 10.39, and stability analysis classified three proteins as stable and 31 as unstable. Based on the aliphatic index and hydrophilicity values, all 34 HSF proteins were determined to be hydrophilic. Subcellular localization predictions suggested that most HSF proteins were targeted to the nucleus, with smaller numbers localized in chloroplasts and the cytoplasm. A limited number of proteins were also predicted to reside in peroxisomes, the Golgi apparatus, the endoplasmic reticulum, and mitochondria (Table S6).

### 3.2. Motif, Domain, and Gene Structure Analysis of Six Transcription Factors Members

**Figure 1:**
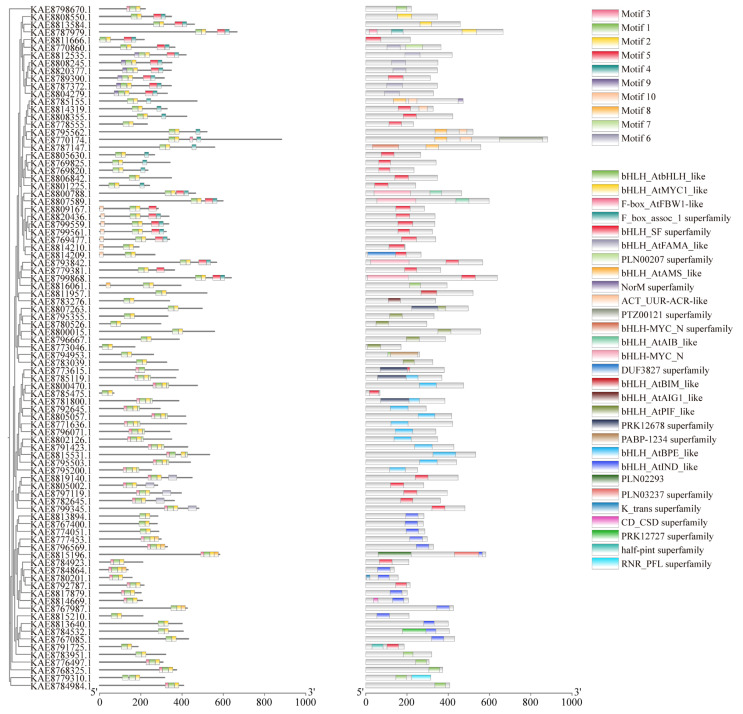
Phylogenetic tree, conserved motifs, and conserved domains of bHLH gene family in highland barley.

Conserved motif analysis of highland barley bHLH proteins via the MEME tool identified a total of 10 conserved motifs. The number of motifs within the bHLH gene family generally ranges from 2 to 5. All bHLH protein sequences exhibit motifs 1 and 2, which are highly conserved and distributed in tandem among all family members. Therefore, it is speculated that motifs 1 and 2 represent conserved motifs of bHLH transcription factors in young seedlings. Domain analysis revealed that bHLH proteins possess a diverse range of domains, with varying numbers of bHLH_SF superfamily domains and other different types of domains. Most bHLH genes, such as *KAE8811666.1* and *KAE8789390.1*, contain bHLH_SF superfamily domains. Additionally, some bHLH proteins possess one or more other types of domains beyond the bHLH_SF superfamily domain. For instance, *KAE8784532.1* includes bHLH_AtINDS-like and PRK12727 superfamily domains, while *KAE8792645.1* features the bHLH_AtBPE-like domain (Figure 1). Visualization of the gene structures of bHLHs using TBtools revealed that members of the bHLH family contain 1 to 12 exons, with 7 members containing only a single exon. Notably, some bHLH genes had longer introns, particularly *bHLH7*, *bHLH40*, and *bHLH82* (Figure S2A).

Conserved motif analysis of highland barley MYB proteins identified a total of 10 conserved motifs. Generally, the number of motifs within the gene family ranges from 1 to 6, with 14 genes exhibiting only 1 motif. Notably, Motif 2 and Motif 5 are highly conserved and present in tandem in most members, suggesting that these two motifs play important roles in the evolutionary development of the MYB gene family in highland barley. Domain analysis indicated that MYB family members have varying numbers of myb_SHAQKYF domains, as well as other different types of domains, highlighting the diversity of MYB protein domains. Specifically, 45 MYB genes possess the myb_SHAQKYF domain, and the majority of members also contain the PLN03091 superfamily domain (Figure S1A). Visualization of the gene structure of MYB using TBtools revealed that MYB genes contain between 1 and 13 exons, with 27 members consisting of only a single exon. Additionally, the *MYB79* and *MYB75* genes are characterized by longer introns (Figure S2B).

MEME motif prediction analysis of the NAC family identified 10 conserved motifs in NAC proteins, with family members typically containing 1 to 8 motifs. Notably, Motif 1 is present in all members, suggesting that it is a crucial component in the evolutionary development of the NAC gene family in highland barley. Furthermore, the majority of NAC gene family members share similar motifs, indicating a potential conservation of function among them. In the domain analysis, all members, with the exception of *KAE8799140.1*, possess typical NAM domains, while certain family members have also evolved to include conserved domains from the PRK10856 and PRK10263 superfamilies (Figure S1B). Visualization of the gene structure of the NAC family demonstrated that all 141 NAC genes exhibit varying numbers of exons. Notably, *NAC20* has the highest exon count at 15, while 34 genes in the family are devoid of introns (Figure S2C).

**Figure 2:**
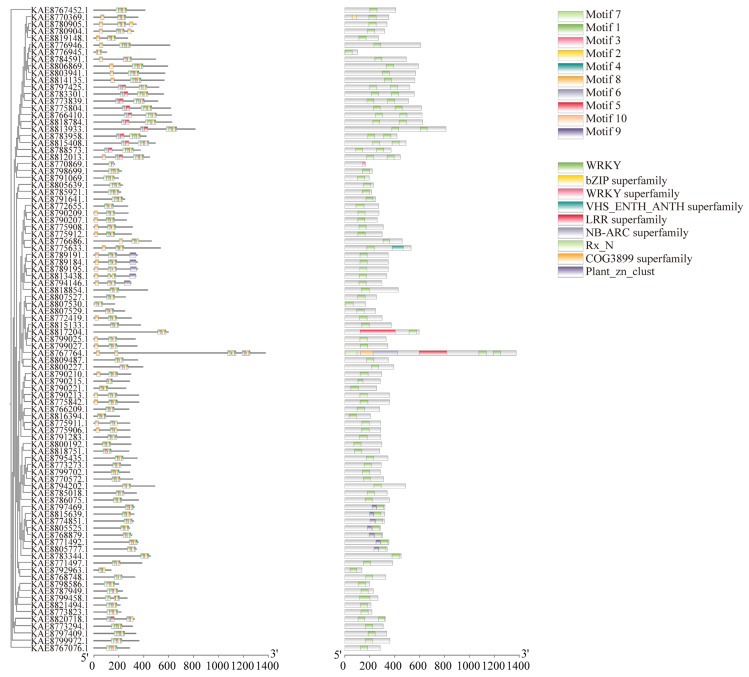
Phylogenetic tree, conserved motifs, and conserved domains of WRKY gene family in highland barley.

The prediction of conserved motifs in the WRKY protein of highland barley identified ten conserved motifs. Generally, the number of motifs in WRKY family members ranges from 2 to 10. A total of 87 genes contain Motifs 1, 2, and 7, including *KAE8789191.1*, *KAE8789184.1*, *KAE8789195.1*, *KAE8813438.1*, and *KAE8794146.1*. Motif 9 is present in all these proteins, suggesting that they share similar functions. In the domain analysis, all WRKY proteins, except for *KAE8770869.1*, which lacks the WRKY superfamily domain, contain a conserved WRKY domain. Additionally, *KAE8805777.1*, *KAE8771492.1*, *KAE8768879.1*, *KAE8805525.1*, *KAE8774851.1*, *KAE8815639.1*, and *KAE8797469.1* also contain the Plant_zn_club conserved domain, indicating its significance as a component of WRKY proteins. Some WRKY proteins possess unique conserved domains; for instance, *KAE8770369.1* contains the bZIP superfamily conserved domain, while *KAE8767764.1* contains the COG3899 superfamily conserved domain (Figure 2). Analysis of the WRKY gene structures revealed that among the 92 WRKY family genes, five members lack introns, while the remaining members have exon-intron structures (Figure S2D).

**Figure 3:**
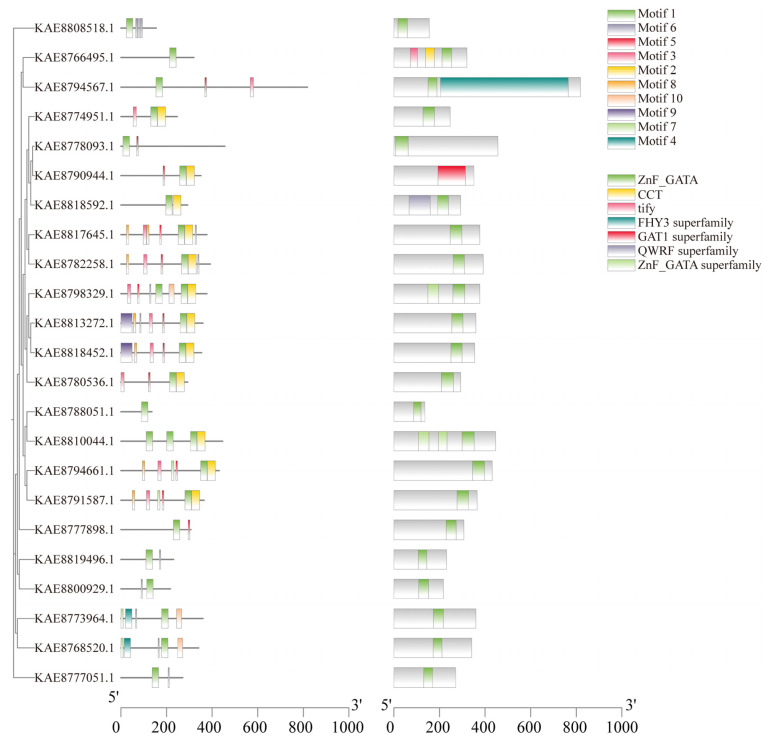
Phylogenetic tree, conserved motifs, and conserved domains of GATA gene family in highland barley.

Conserved motif prediction of GATA proteins identified a total of 10 conserved motifs. The number of motifs present in members of the gene family generally ranges from 1 to 7. Motif 1 is found in all GATA protein sequences, indicating its significance as a key component in the evolutionary development of the GATA gene family in highland barley. Notably, 12 genes possess Motif 2, while *KAE8813272.1* and *KAE8818452.1* contain Motif 9, suggesting that these proteins may share similar functions. Conserved domain analysis indicated that, except for *KAE8790944.1* (which contains the GAT1 superfamily domain), all other 22 members possess the ZnF_GATA domain. Furthermore, *KAE8766495.1* also includes CCT and TIFY domains, *KAE8794567.1* contains the FHY3 superfamily domain, and *KAE8818592.1* features the QWRF superfamily domain (Figure 3). Visualization of the gene structures of GATA genes using TBtools revealed that the number of exons ranges from 1 to 8, with 4 members containing only one exon and 4 members lacking introns (Figure S2E).

**Figure 4:**
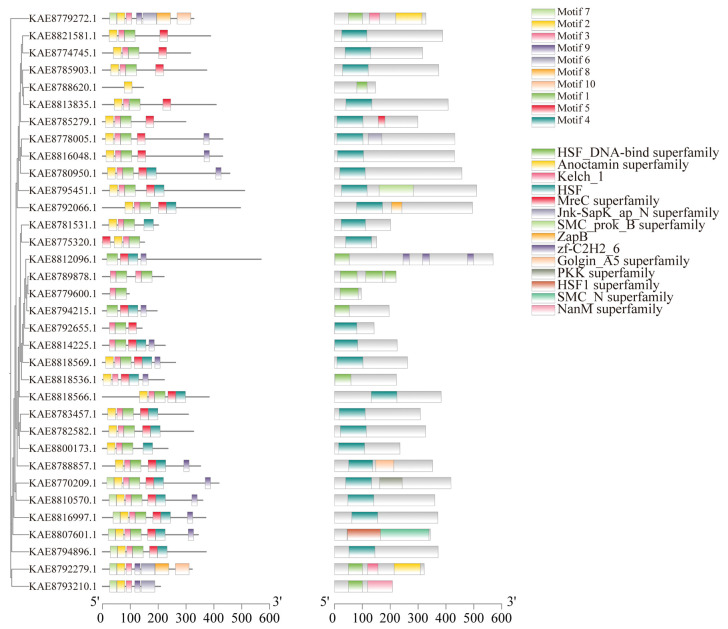
Phylogenetic tree, conserved motifs, and conserved domains of HSF gene family in highland barley.

Conserved motif prediction for HSF proteins in highland barley identified a total of 10 conserved motifs, with most members of the HSF gene family containing 2 to 7 motifs. Motif 2 is present in all 28 HSF protein sequences, and Motif 1 is present in all 29 HSF protein sequences. It is speculated that Motif 1 and Motif 2 are important components of HSF transcription factors in highland barley seedlings. Motif 7 appears in 8 members, and Motif5 appears in 26 members, suggesting that HSF proteins with similar motif structures and functions are also similar among HSF members. Structural prediction of HSF proteins encoded by members of the highland barley HSF gene family showed that 24 out of 34 HSF family members contain HSF domains, *KAE8807601.1* contains the HSF1 superfamily domain, and 9 members contain the HSF1 DNA-binding superfamily domain. In addition, a small number of members contain other domains, such as *KAE8812096.1* containing the zf-C2H2-6 domain and *KAE8792066.1* containing the ZapB domain. (Figure 4). In the analysis of gene structure, it was found that among the 34 members of the HSF gene family in highland barley, there are 1–8 exons, and 9 genes only contain 1 exon. Among them, *HSF4* contains a longer intron (Figure S2F).

### 3.3. Analysis of Cis-Acting Elements in Gene Families of Transcription Factor Members

Cis-acting elements in promoters, as key binding sites for transcription factors, play a pivotal role in regulating gene expression. In this study, the online PlantCARE tool was used to analyze the 2000 bp upstream sequences of the start codons of genes from the six transcription factor families (bHLH, MYB, NAC, GATA, WRKY, and HSF) in highland barley seedlings. The identified cis-acting elements were then visualized using TBtools, which revealed 34, 41, 41, 30, 33, and 29 distinct elements in the bHLH, MYB, NAC, GATA, WRKY, and HSF families, respectively. These cis-acting elements were classified into five functional categories: light response, hormone response, plant development, stress response, and other functions. Notably, light-responsive elements were the most abundant and widely distributed across all six families, predominantly including canonical light-responsive elements and their derivative variants. Furthermore, a substantial number of hormone-responsive elements were detected in the promoter regions of these families, such as auxin-responsive elements (AuxRE, TGA element), gibberellin-responsive elements (GARE motif, P-box, TATC box), jasmonic acid-responsive elements (CGTCA motif, TGACG motif), abscisic acid-responsive elements (ABRE), and salicylic acid-responsive elements (TCA element). In addition to light and hormone-responsive elements, multiple stress-related cis-acting elements were identified, including drought-responsive MYB-binding sites (MBS), enhancer-like elements involved in anoxic-specific induction, low-temperature-responsive elements (LTR), and TC-rich repeats associated with stress defense. Additionally, numerous cis-acting elements implicated in plant development were characterized, such as the seed development regulatory RY element, endosperm-specific expression GCN4 motif, and meristem-specific expression CAT box. MSA-like cell cycle regulatory elements were found in the GATA, NAC, MYB, and WRKY families, while HD Zip elements associated with palisade mesophyll cell differentiation were detected in the GATA, NAC, and MYB families. Circadian rhythm regulatory elements were also identified across the families. Collectively, these findings suggest that genes from the bHLH, MYB, NAC, GATA, WRKY, and HSF families may play significant roles in mediating the response of highland barley seedlings to environmental stresses, as well as regulating their growth and developmental processes. (Figure S3A–F).

### 3.4. Phylogenetic Tree Analysis of Transcription Factors Members

To elucidate the evolutionary relationship among six families of proteins in highland barley, we performed multiple sequence alignments with wheat proteins and constructed a phylogenetic tree. The results indicated that the transcription factors of the bHLH, MYB, NAC, WRKY, GATA, and HSF families are classified into 9, 8, 9, 5, 6, and 5 subfamilies, respectively. Notably, the Clade I subfamily contains the largest number of members, with 26, 65, 64, 41, 12, and 13 members from the highland barley bHLH, MYB, NAC, WRKY, GATA, and HSF families, respectively. Genes clustered within the same branch exhibit close genetic relationships, and gene structures within the same subfamily show similarities. The variation in the number of members across each family suggests that they share similar evolutionary trends and may perform analogous biological functions (Figure S4A–F).

### 3.5. Gene Expression Patterns of Six Transcription Factor Members

**Figure 5:**
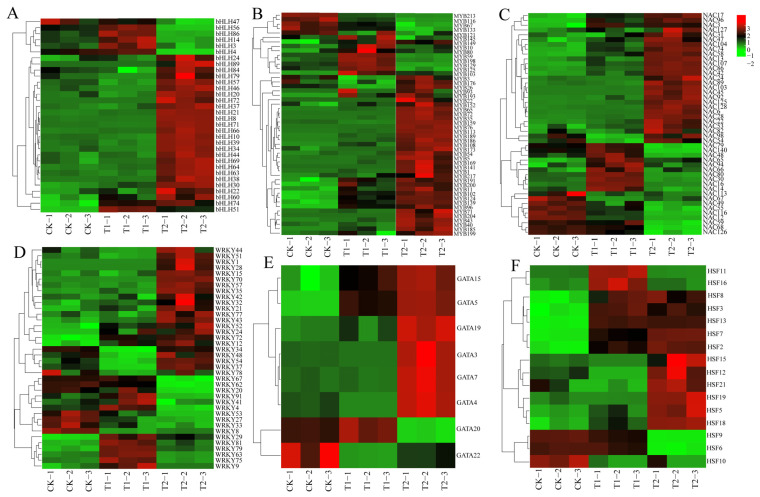
Expression analysis of 6 family genes under different concentrations of Na_2_SeO_3_: (**A**) bHLH family, (**B**) MYB family, (**C**) NAC family, (**D**) WRKY family, (**E**) GATA family, (**F**) HSF family. T1: 0.02 g/kg Na_2_SeO_3_ treatment; T2: 0.2 g/kg Na_2_SeO_3_ treatment.

To investigate the expression characteristics of six highland barley gene families under different sodium selenite treatments, we utilized the transcriptome data previously obtained by our research group. The FPKM normalization method (Table S7) was adopted to quantitatively analyze the expression levels of significantly differential members in the selected six gene families, and an expression heatmap was generated (Figure 5). Under T1 treatment, 56.25% of the genes in the bHLH family were significantly upregulated, whereas approximately 81.25% of the genes were significantly upregulated under T2 treatment (Figure 5A). In the MYB family, 76% of the genes were significantly upregulated under T1 treatment, while about 80% of the genes were significantly upregulated under T2 treatment (Figure 5B). For the NAC family, 67.39% of the genes exhibited significant upregulation under T1 treatment, whereas approximately 65.22% of the genes were significantly upregulated under T2 treatment (Figure 5C). In the WRKY family, 47.37% of the genes showed significant upregulation in their expression under T1 treatment, while about 63.16% of the genes had significantly upregulated expression under T2 treatment (Figure 5D). In the GATA family, 50% of the genes were significantly upregulated under T1 treatment, and approximately 75% of the genes were significantly upregulated under T2 treatment (Figure 5E). Finally, in the HSF family, 56.25% of the genes were significantly upregulated under T1 treatment, whereas about 75% of the genes were significantly upregulated under T2 treatment (Figure 5F).

Compared with the control group (CK), the expression levels of bHLH71, bHLH10, and bHLH30 peaked under high-concentration treatment, showing significant upregulation by 20.64-fold, 5.29-fold, and 1.24-fold, respectively. Similarly, MYB96, MYB43, and MYB65 also reached their highest expression levels under high-concentration treatment, with respective increases of 1.70-fold, 3.87-fold, and 2.44-fold. For the NAC family, NAC128, NAC86, and NAC107 exhibited peak expression under high-concentration treatment, showing significant upregulation by 4.11-fold, 1.27-fold, and 29-fold, respectively. In addition, WRKY57, WRKY72, and WRKY37 reached their expression maxima under high-concentration treatment, with increases of 1.31-fold, 1.36-fold, and 0.36-fold, respectively. Likewise, GATA7, GATA15, and GATA3 showed peak expression under high-concentration treatment, with substantial upregulation of 2.94-fold, 1.75-fold, and 20.67-fold, respectively. Finally, HSF13, HSF7, and HSF8 attained their highest expression levels under high-concentration treatment, with significant increases of 10.2-fold, 3-fold, and 1.87-fold, respectively. However, selenium treatment inhibited the expression levels of certain members, including *MYB213*, *MYB116*, *NAC113*, *NAC67*, *NAC49*, *WRKY8*, *WRKY33*, *WRKY27*, *WRKY53*, *GATA22*, *HSF10*, *HSF6*, and *HSF9*.

### 3.6. qRT-PCR Validation of Transcription Factors of Highland Barley Seedlings Under Na_2_SeO_3_ Treatment

**Figure 6:**
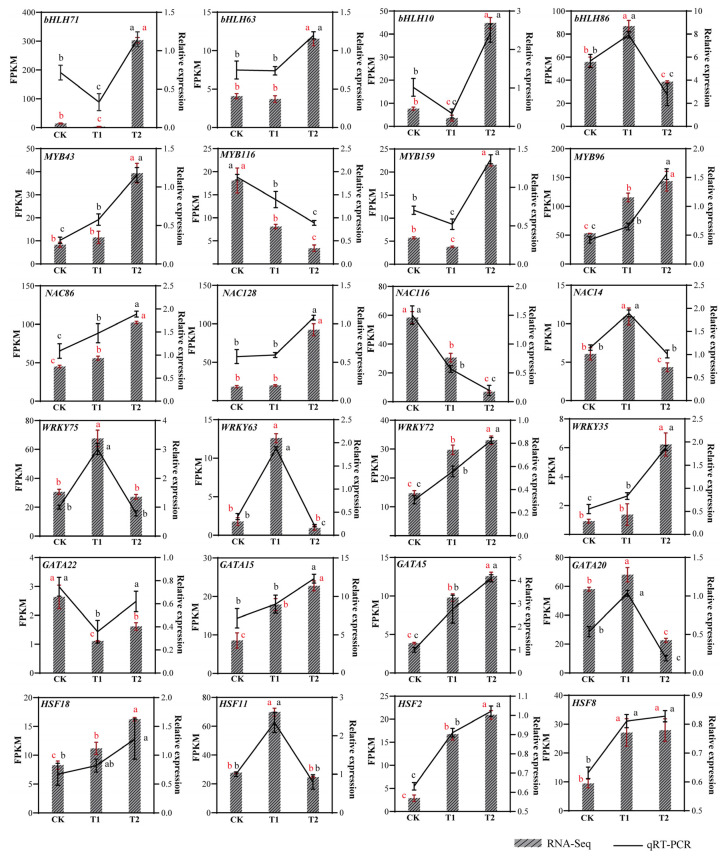
Comprehensive transcriptome analysis and qRT-PCR validation were performed to examine differentially expressed transcription factors across the treatment groups. Relative expression was calculated using the 2^−ΔΔCt^ method. The red vertical bar represents the mean ± SD of the three repetitions of FPKM. The black vertical bar represents the mean ± SD of the three repeats of qRT-PCR. (a, b, c) indicate significant differences between groups: the same letters indicate no significant nificant difference between groups, while different letters indicate significant differences between groups. The red letter represents the significance of FPKM, and the black letter represents the significance of qRT-PCR. CK: control; T1: 0.02 g/kg Na_2_SeO_3_ treatment; T2: 0.2 g/kg Na_2_SeO_3_ treatment.

In this study, we selected four differentially expressed transcription factor families from each family for expression level validation (Figure 6). Using the EF-1α housekeeping gene as an internal reference, we standardized the expression levels of target genes. Primer sequences for each gene are listed in Table S8. The results indicated that the expression levels of MYB43, MYB96, NAC86, WRKY72, WRKY35, GATA15, GATA5, HSF18, HSF2 and HSF8 genes increased with higher concentrations of Na_2_SeO_3_ treatment. Conversely, the expression levels of MYB116 and NAC116 decreased with increasing Na_2_SeO_3_ concentration. bHLH86, NAC14, WRKY75, WRKY63, GATA20, and HSF11 have the highest expression levels at low selenium concentrations. The trends observed in the qRT-PCR results for 24 genes were consistent with the transcriptome expression trends. These outcomes demonstrate that the transcriptome expression data are reliable and accurate.

## 4. Discussion

Transcription factors (TFs) are key regulatory factors that coordinate various biological processes by activating or repressing the expression of target genes [58,59,60,61,62]. Currently, NAC, MYB, WRKY, bHLH, and AP2/ERF are among the most widely studied transcription factors. These transcription factors regulate the expression of target genes by binding to cis-acting elements in promoter regions, and their significance in mediating plant responses to abiotic and biotic stresses is well recognized [63,64]. With the continuous advancement of genomic sequencing technologies, many researchers have systematically identified and analyzed members of transcription factor gene families in various plants. To date, transcription factor gene families have been extensively identified across diverse plant species; for instance, 208 bHLH family members have been reported in *Zea mays* [65], and 602 bHLH members have been identified in *Brassica napus* [66]. In *Arabidopsis thaliana*, 198 MYB members have been identified [67], while 185 MYB members have been identified in *Oryza sativa* [68]. Thirty GATA genes were identified in *Arabidopsis thaliana* [69] and twenty-eight GATA genes in *Brachypodium distachyon* [70]. Additionally, different numbers of WRKY members were identified in *Solanum lycopersicum* [71], *Carica papaya* [72], and cotton [73]. These gene families are widely involved in various signal transduction and metabolic pathways. This study identified six transcription factor families in the seedlings of hulless highland barley, including bHLH (89), MYB (218), NAC (141), WRKY (92), GATA (23), and HSF (34). The differences in gene numbers compared to other species may be related to varying degrees of gene amplification in different plants. Physicochemical property analysis indicated that most proteins in these families are hydrophilic and primarily localized in the nucleus—an organelle that stores substantial genetic information and serves as a key site for DNA replication and transcription—consistent with previous findings in *Brassica napus* [66]. Research indicates that most HSF family proteins are hydrophilic and exhibit instability—for example, 17 HSF members in carnations are hydrophilic and have low stability [74].

Structural analysis indicated that each family contains highly conserved motifs and domains, such as the bHLH_SF superfamily domain of the bHLH family, the myb_SHAQKYF domain of the MYB family, and the NAM domain of the NAC family. These conserved structures are core components of their transcriptional regulatory functions, and the exon-intron structures along with the conserved motifs are highly consistent within subgroups, aligning with typical family characteristics. Changes in gene structure and their conserved motifs reflect the evolutionary, differentiation, conservation, and functional divergence characteristics of gene families [75]. Some genes exhibit variations in the number of motifs among individual family members, such as the duplication of motifs in *KAE8810044.1* and *KAE8798329.1*, indicating their potential functional specialization. Cis-acting elements contribute to the regulation of stress-responsive gene expression in organisms [76]. Genes from the six transcription factor families have promoter regions rich in light-, hormone-, and stress-responsive elements, and light-responsive elements are the most prevalent. Studies have shown that the cis-acting elements involved in plant growth and development are part of conserved DNA modules that participate in light responses [77]. In our study, phylogenetic analysis indicated that the bHLH, MYB, NAC, WRKY, GATA, and HSF transcription factors can be categorized into 9, 8, 9, 5, 6, and 5 subfamilies, respectively, based on the evolutionary tree constructed with wheat homologs. Clade I is the most abundant and core subfamily across all six families. Clade I may represent the most conserved and core branch of these six transcription factor families, preserved throughout the long evolutionary history of highland barley, suggesting its potential role in the polyploidization of highland barley. Polyploid plants exhibit richer biomass and stronger stress resistance compared to diploid plants [78]. Therefore, members of these transcription factor families in highland barley may have greater advantages in promoting plant growth and development or coping with environmental adversity.

To better understand the role of transcription factors, expression analysis was conducted under different concentrations of Na_2_SeO_3_ based on the preliminary data from our research group. Studies on maize seedlings indicated that high selenium treatment significantly upregulated genes such as WRKY to promote the detoxification of selenium compounds [45]. Research on aloe vera demonstrated that after selenium treatment, the heat stress transcription factors *A-2b* and *TFIID* were significantly upregulated at T1 (200 mg/L) and T2 (400 mg/L). The transcription levels of MYB, bHLH, GATA, and IBH1 family genes are higher at T2 than at T1, and selenium treatment can strongly induce the transcription of heat shock proteins, HEX, HY5, and PosF21, particularly under T2 [79]. Integration of transcriptome data and selenium content indicated that 12 BpNAC genes were related to the biosynthesis of selenium [80]. In our study, transcriptome data analysis revealed differential expression patterns of the six transcription factor families under different concentrations of sodium selenite treatment, with most genes significantly upregulated under high selenium treatment. Genes such as *bHLH71*, *MYB96*, *NAC128*, *WRKY57*, *GATA7*, and *HSF13* reached peak expression levels under high concentrations of selenium treatment. Notably, the expression level of *bHLH71* increased significantly by 20.64 times compared to the control group. High levels of selenium may induce chronic moderate toxicity in plants, resulting in reduced biomass and inhibited growth [81]. Research on corn has shown that ZmWRKY48 has been identified as a central regulatory factor for selenium metabolism and detoxification [45]. In our research, the expression of certain genes including MYB213, NAC113, WRKY8, and GATA22, was suppressed under selenium treatment, indicating that there may be a synergistic regulatory mechanism among transcription factor families that balances gene expression to mitigate damage caused by excessive stress responses in plants. Furthermore, the relative expression levels of genes under high concentration treatment (T2, 0.2 g/kg) were significantly higher than those under low concentration treatment (T1, 0.02 g/kg), demonstrating that highland barley’s response to selenium stress is concentration-dependent, further confirming the concentration effect characteristic of abiotic stress responses.

This study systematically reveals the molecular response characteristics of six transcription factor families in highland barley under selenium stress, laying a solid theoretical foundation and providing valuable genetic resources for future research. In the future, techniques such as gene editing and multi-omics integrated analysis can be employed to deeply dissect the regulatory mechanisms of core genes like bHLH71 and MYB43, exploring their specific functions in selenium metabolism and antioxidant stress, which will offer novel targets for the molecular breeding of stress-tolerant highland barley. Furthermore, our findings not only fill the gap in studies on transcriptional regulation of selenium stress responses in plateau crops and enrich the theoretical system of plant stress responses but also promote the innovation of stress-tolerant germplasm resources for characteristic plateau crops and the sustainable development of ecological agriculture, thus serving as a reference model for stress response research on similar plateau crops.

## 5. Conclusions

This study characterized members of six transcription factor families (bHLH, MYB, NAC, WRKY, GATA, and HSF) in highland barley seedlings using bioinformatics analyses, clarifying their physicochemical properties, gene structures, conserved domains, phylogenetic relationships, and cis-acting element characteristics. By integrating transcriptome data, we further revealed the expression patterns of these transcription factors in response to selenium stress. The results showed that most proteins from these six families are hydrophilic nuclear proteins, harboring conserved domains and multiple response elements in their promoter regions. These structural traits lay the foundation for their involvement in plant growth, development, and stress responses. Notably, the number of upregulated genes in these six families was significantly higher under high selenium concentration (T2) than under low selenium concentration (T1), indicating that highland barley’s transcriptional response to selenium stress is concentration-dependent. Core genes such as bHLH71, MYB43, and GATA3 may play key roles in selenium stress tolerance, while the suppressed expression of genes including MYB116 and WRKY63 may participate in balancing plant stress responses. Overall, highland barley’s transcriptional response to selenium stress varies with selenium concentration, representing a comprehensive adaptive strategy that activates stress resistance mechanisms while alleviating selenium toxicity. This study is the first to systematically elucidate the molecular characteristics of these six transcription factor families in highland barley and their responses to selenium stress, providing a fundamental theoretical basis for a deeper understanding of the molecular regulatory mechanisms underlying highland barley’s adaptation to selenium stress.

## Data Availability

Raw RNA-seq data have been deposited in the NCBI SRA database with the project number PRJNA1266111, accessible online at https://dataview.ncbi.nlm.nih.gov/object/PRJNA1266111?reviewer=bmhd9p8e0vrrap3tllce83v243, accessed on 1 December 2024.

## Supplementary Materials

The following supporting information can be downloaded at: https://www.mdpi.com/article/10.3390/life16020255/s1, Figure S1 Phylogenetic tree, conserved motifs, and conserved domains of gene family in highland barley (A) MYB. (B) NAC; Figure S2 Gene structure of highland barley transcription factor family members. (A) bHLH. (B) MYB. (C) NAC. (D) WRKY. (E) GATA. (F) HSF; Figure S3 Analysis of cis-acting elements of highland barley transcription factor family members. (A) bHLH. (B) MYB. (C) NAC. (D) WRKY. (E) GATA. (F) HSF; Figure S4 Phylogenetic tree of transcription factor genes in highland barley and wheat. (A) bHLH. (B) MYB. (C) NAC. (D) WRKY. (E) GATA. (F) HSF. Used IQ tree to construct the evolutionary tree using the maximum likelihood method (bootstrap value: 1000 repetitions); Table S1: Proteins features of bHLH TF family members and the subcellular localizations; Table S2: Proteins features of MYB TF family members and the subcellular localizations; Table S3: Proteins features of NAC TF family members and the subcellular localizations; Table S4: Proteins features of WRKY TF family members and the subcellular localizations; Table S5: Proteins features of GATA TF family members and the subcellular localizations; Table S6: Proteins features of HSF TF family members and the subcellular localizations; Table S7: Differential gene expression levels of six transcription factor families; Table S8: The primers used for RT-qPCR and Transcript ID of 24 genes.

## Abbreviations

The following abbreviations are used in this manuscript:

bHLH	Basic helix–loop–helix
MYB	v-myb avian myeloblastosis viral oncogene homolog
NAC	NAM, ATAF1/2 and CUC2
WRKY	WRKY DNA-binding protein
GATA	GATA-binding protein
HSF	Heat stress transcription factor

## References

[1] Zhang C., Feng R., Ma R., Shen Z., Cai Z., Song Z., Peng B., Yu M. (2018). Genome-Wide Analysis of Basic Helix-Loop-Helix Superfamily Members in Peach. PLoS ONE.

[2] Feller A., Machemer K., Braun E.L., Grotewold E. (2011). Evolutionary and Comparative Analysis of MYB and bHLH Plant Transcription Factors. Plant J..

[3] Ludwig S.R., Habera L.F., Dellaporta S.L., Wessler S.R. (1989). Lc, a Member of the Maize R Gene Family Responsible for Tissue-Specific Anthocyanin Production, Encodes a Protein Similar to Transcriptional Activators and Contains the Myc-Homology Region. Proc. Natl. Acad. Sci. USA.

[4] Toledo-Ortiz G., Huq E., Quail P.H. (2003). The Arabidopsis Basic/Helix-Loop-Helix Transcription Factor Family. Plant Cell.

[5] Heim M.A., Jakoby M., Werber M., Martin C., Weisshaar B., Bailey P.C. (2003). The Basic Helix-Loop-Helix Transcription Factor Family in Plants: A Genome-Wide Study of Protein Structure and Functional Diversity. Mol. Biol. Evol..

[6] Murre C., McCaw P.S., Baltimore D. (1989). A New DNA Binding and Dimerization Motif in Immunoglobulin Enhancer Binding, Daughterless, MyoD, and Myc Proteins. Cell.

[7] Pires N., Dolan L. (2010). Origin and Diversification of Basic-Helix-Loop-Helix Proteins in Plants. Mol. Biol. Evol..

[8] Martin C., Paz-Ares J. (1997). MYB Transcription Factors in Plants. Trends Genet..

[9] Du H., Zhang L., Liu L., Tang X.-F., Yang W.-J., Wu Y.-M., Huang Y.-B., Tang Y.-X. (2009). Biochemical and Molecular Characterization of Plant MYB Transcription Factor Family. Biochemistry.

[10] Olsen A.N., Ernst H.A., Leggio L.L., Skriver K. (2005). NAC Transcription Factors: Structurally Distinct, Functionally Diverse. Trends Plant Sci..

[11] Li C., Zhang J., Zhang Q., Dong A., Wu Q., Zhu X., Zhu X. (2022). Genome-Wide Identification and Analysis of the NAC Transcription Factor Gene Family in Garden Asparagus (*Asparagus officinalis*). Genes.

[12] Hu X., Xie F., Liang W., Liang Y., Zhang Z., Zhao J., Hu G., Qin Y. (2022). HuNAC20 and HuNAC25, Two Novel NAC Genes from Pitaya, Confer Cold Tolerance in Transgenic Arabidopsis. Int. J. Mol. Sci..

[13] Hu H., Ma L., Chen X., Fei X., He B., Luo Y., Liu Y., Wei A. (2022). Genome-Wide Identification of the NAC Gene Family in *Zanthoxylum bungeanum* and Their Transcriptional Responses to Drought Stress. Int. J. Mol. Sci..

[14] Xie Z., Zhang Z.-L., Zou X., Huang J., Ruas P., Thompson D., Shen Q.J. (2005). Annotations and Functional Analyses of the Rice WRKY Gene Superfamily Reveal Positive and Negative Regulators of Abscisic Acid Signaling in Aleurone Cells. Plant Physiol..

[15] Chen X., Li C., Wang H., Guo Z. (2019). WRKY Transcription Factors: Evolution, Binding, and Action. Phytopathol. Res..

[16] Hannon R., Evans T., Felsenfeld G., Gould H. (1991). Structure and Promoter Activity of the Gene for the Erythroid Transcription Factor GATA-1. Proc. Natl. Acad. Sci. USA.

[17] Lowry J.A., Atchley W.R. (2000). Molecular Evolution of the GATA Family of Transcription Factors: Conservation within the DNA-Binding Domain. J. Mol. Evol..

[18] Reyes J.C., Muro-Pastor M.I., Florencio F.J. (2004). The GATA Family of Transcription Factors in Arabidopsis and Rice. Plant Physiol..

[19] Daniel-Vedele F., Caboche M. (1993). A Tobacco cDNA Clone Encoding a GATA-1 Zinc Finger Protein Homologous to Regulators of Nitrogen Metabolism in Fungi. Mol. Gen. Genet..

[20] Duan S., Liu B., Zhang Y., Li G., Guo X. (2019). Genome-Wide Identification and Abiotic Stress-Responsive Pattern of Heat Shock Transcription Factor Family in *Triticum aestivum* L.. BMC Genom..

[21] Reddy P.S., Kavi Kishor P.B., Seiler C., Kuhlmann M., Eschen-Lippold L., Lee J., Reddy M.K., Sreenivasulu N. (2014). Unraveling Regulation of the Small Heat Shock Proteins by the Heat Shock Factor HvHsfB2c in Barley: Its Implications in Drought Stress Response and Seed Development. PLoS ONE.

[22] Jiang L., Hu W., Qian Y., Ren Q., Zhang J. (2021). Genome-Wide Identification, Classification and Expression Analysis of the Hsf and Hsp70 Gene Families in Maize. Gene.

[23] Guo M., Lu J.-P., Zhai Y.-F., Chai W.-G., Gong Z.-H., Lu M.-H. (2015). Genome-Wide Analysis, Expression Profile of Heat Shock Factor Gene Family (CaHsfs) and Characterisation of CaHsfA2 in Pepper (*Capsicum annuum* L.). BMC Plant Biol..

[24] Harrison C.J., Bohm A.A., Nelson H.C. (1994). Crystal Structure of the DNA Binding Domain of the Heat Shock Transcription Factor. Science.

[25] Li C., Li Y., Zhou Z., Huang Y., Tu Z., Zhuo X., Tian D., Liu Y., Di H., Lin Z. (2023). Genome-Wide Identification and Comprehensive Analysis Heat Shock Transcription Factor (Hsf) Members in Asparagus (*Asparagus officinalis*) at the Seeding Stage under Abiotic Stresses. Sci. Rep..

[26] Han D., Lai J., Yang C. (2021). SUMOylation: A Critical Transcription Modulator in Plant Cells. Plant Sci..

[27] Khan I., Khan S., Zhang Y., Zhou J., Akhoundian M., Jan S.A. (2021). CRISPR-Cas Technology Based Genome Editing for Modification of Salinity Stress Tolerance Responses in Rice (*Oryza sativa* L.). Mol. Biol. Rep..

[28] Gupta P., Nutan K.K., Singla-Pareek S.L., Pareek A. (2017). Abiotic Stresses Cause Differential Regulation of Alternative Splice Forms of GATA Transcription Factor in Rice. Front. Plant Sci..

[29] Sun K., Wang H., Xia Z. (2019). The Maize bHLH Transcription Factor bHLH105 Confers Manganese Tolerance in Transgenic Tobacco. Plant Sci..

[30] Song Y., Li S., Sui Y., Zheng H., Han G., Sun X., Yang W., Wang H., Zhuang K., Kong F. (2022). SbbHLH85, a bHLH Member, Modulates Resilience to Salt Stress by Regulating Root Hair Growth in Sorghum. Theor. Appl. Genet..

[31] Fang Y., Liu J., Zheng M., Zhu S., Pei T., Cui M., Chang L., Xiao H., Yang J., Martin C. (2023). SbMYB3 Transcription Factor Promotes Root-Specific Flavone Biosynthesis in *Scutellaria baicalensis*. Hortic. Res..

[32] Zhao T., Wu T., Pei T., Wang Z., Yang H., Jiang J., Zhang H., Chen X., Li J., Xu X. (2021). Overexpression of SlGATA17 Promotes Drought Tolerance in Transgenic Tomato Plants by Enhancing Activation of the Phenylpropanoid Biosynthetic Pathway. Front. Plant Sci..

[33] Zhou Y., Wang Y., Xu F., Song C., Yang X., Zhang Z., Yi M., Ma N., Zhou X., He J. (2022). Small HSPs Play an Important Role in Crosstalk between HSF-HSP and ROS Pathways in Heat Stress Response through Transcriptomic Analysis in Lilies (*Lilium longiflorum*). BMC Plant Biol..

[34] Shah Z., Iqbal A., Khan F.U., Khan H.U., Durrani F., Ahmad M.Z. (2020). Genetic Manipulation of Pea (*Pisum sativum* L.) with Arabidopsis’s Heat Shock Factor HsfA1d Improves ROS Scavenging System to Confront Thermal Stress. Genet. Resour. Crop Evol..

[35] Zandalinas S.I., Fritschi F.B., Mittler R. (2020). Signal Transduction Networks during Stress Combination. J. Exp. Bot..

[36] Shah W.H., Rasool A., Saleem S., Mushtaq N.U., Tahir I., Hakeem K.R., Rehman R.U. (2021). Understanding the Integrated Pathways and Mechanisms of Transporters, Protein Kinases, and Transcription Factors in Plants under Salt Stress. Int. J. Genom..

[37] Du X., Wang G., Ji J., Shi L., Guan C., Jin C. (2017). Comparative Transcriptome Analysis of Transcription Factors in Different Maize Varieties under Salt Stress Conditions. Plant Growth Regul..

[38] Xiang L., Wang M., Huang J., Jiang W., Yan Z., Chen X., Yin C., Mao Z. (2022). MdWRKY74 Is Involved in Resistance Response to Apple Replant Disease. Plant Growth Regul..

[39] Wu X., Tao M., Meng Y., Zhu X., Qian L., Shah A., Wang W., Cao S. (2020). The Role of WRKY47 Gene in Regulating Selenium Tolerance in *Arabidopsis thaliana*. Plant Biotechnol. Rep..

[40] Zeng X., Guo Y., Xu Q., Mascher M., Guo G., Li S., Mao L., Liu Q., Xia Z., Zhou J. (2018). Origin and Evolution of Qingke Barley in Tibet. Nat. Commun..

[41] Xie J., Hong Y., Gu Z., Cheng L., Li Z., Li C., Ban X. (2023). Highland Barley Starch: Structures, Properties, and Applications. Foods.

[42] Liang J., Chen X., Deng G., Pan Z., Zhang H., Li Q., Yang K., Long H., Yu M. (2017). Dehydration Induced Transcriptomic Responses in Two Tibetan Hulless Barley (*Hordeum vulgare* var. *nudum*) Accessions Distinguished by Drought Tolerance. BMC Genom..

[43] Zhu F., Du B., Xu B. (2016). A Critical Review on Production and Industrial Applications of Beta-Glucans. Food Hydrocoll..

[44] Wu F., Li W., Liu X., Li W., Guo X., Zhao Y., Zhang H., Song Q., Liu F., Zhang P. (2025). Se Improves Cd Tolerance by Modulating Phytohormone Signaling and Primary Metabolism in Strawberry. J. Hazard. Mater..

[45] Wang R., Zhang H., Liu Z., Lu Y., Lan S., Zhang B., Li S., Li Q., Ma J., Xiang X. (2025). Uncovering the Transcriptional Regulatory Network Underlying Selenium Tolerance in Maize Seedlings. J. Hazard. Mater..

[46] Finn R.D., Coggill P., Eberhardt R.Y., Eddy S.R., Mistry J., Mitchell A.L., Potter S.C., Punta M., Qureshi M., Sangrador-Vegas A. (2016). The Pfam Protein Families Database: Towards a More Sustainable Future. Nucleic Acids Res..

[47] Eddy S.R. (2011). Accelerated Profile HMM Searches. PLoS Comput. Biol..

[48] Marchler-Bauer A., Derbyshire M.K., Gonzales N.R., Lu S., Chitsaz F., Geer L.Y., Geer R.C., He J., Gwadz M., Hurwitz D.I. (2015). CDD: NCBI’s Conserved Domain Database. Nucleic Acids Res..

[49] Wilkins M.R., Gasteiger E., Bairoch A., Sanchez J.C., Williams K.L., Appel R.D., Hochstrasser D.F. (1999). Protein Identification and Analysis Tools in the ExPASy Server. Methods Mol. Biol..

[50] Horton P., Park K.-J., Obayashi T., Fujita N., Harada H., Adams-Collier C.J., Nakai K. (2007). WoLF PSORT: Protein Localization Predictor. Nucleic Acids Res..

[51] Chen C., Wu Y., Li J., Wang X., Zeng Z., Xu J., Liu Y., Feng J., Chen H., He Y. (2023). TBtools-II: A “One for All, All for One” Bioinformatics Platform for Biological Big-Data Mining. Mol. Plant.

[52] Chen C., Chen H., Zhang Y., Thomas H.R., Frank M.H., He Y., Xia R. (2020). TBtools: An Integrative Toolkit Developed for Interactive Analyses of Big Biological Data. Mol. Plant.

[53] Lescot M., Déhais P., Thijs G., Marchal K., Moreau Y., Van de Peer Y., Rouzé P., Rombauts S. (2002). PlantCARE, a Database of Plant Cis-Acting Regulatory Elements and a Portal to Tools for in Silico Analysis of Promoter Sequences. Nucleic Acids Res..

[54] Nguyen L.-T., Schmidt H.A., von Haeseler A., Minh B.Q. (2015). IQ-TREE: A Fast and Effective Stochastic Algorithm for Estimating Maximum-Likelihood Phylogenies. Mol. Biol. Evol..

[55] Letunic I., Bork P. (2021). Interactive Tree Of Life (iTOL) v5: An Online Tool for Phylogenetic Tree Display and Annotation. Nucleic Acids Res..

[56] Wu X., Xie H., Ma J., Geng G., Yang X., Qiao F. (2025). Organic Acids Metabolic Response and Transcription Factor Expression Changes of Highland Barley Seedlings Under Na_2_SeO_3_ Treatment. Agriculture.

[57] Livak K.J., Schmittgen T.D. (2001). Analysis of Relative Gene Expression Data Using Real-Time Quantitative PCR and the 2(-Delta Delta C(T)) Method. Methods.

[58] Capella M., Ribone P.A., Arce A.L., Chan R.L. (2015). Arabidopsis Thaliana HomeoBox 1 (AtHB1), a Homedomain-Leucine Zipper I (HD-Zip I) Transcription Factor, Is Regulated by PHYTOCHROME-INTERACTING FACTOR 1 to Promote Hypocotyl Elongation. New Phytol..

[59] Li Z., Zhang C., Guo Y., Niu W., Wang Y., Xu Y. (2017). Evolution and Expression Analysis Reveal the Potential Role of the HD-Zip Gene Family in Regulation of Embryo Abortion in Grapes (*Vitis vinifera* L.). BMC Genom..

[60] Shi Y., Ding Y., Yang S. (2018). Molecular Regulation of CBF Signaling in Cold Acclimation. Trends Plant Sci..

[61] Hu D.-G., Yu J.-Q., Han P.-L., Xie X.-B., Sun C.-H., Zhang Q.-Y., Wang J.-H., Hao Y.-J. (2019). The Regulatory Module MdPUB29-MdbHLH3 Connects Ethylene Biosynthesis with Fruit Quality in Apple. New Phytol..

[62] Xi W., He Y., Zhu L., Hu S., Xiong S., Zhang Y., Zou J., Chen H., Wang C., Zheng R. (2021). CPTA Treatment Reveals Potential Transcription Factors Associated with Carotenoid Metabolism in Flowers of *Osmanthus fragrans*. Hortic. Plant J..

[63] Xu X., Mo Q., Cai Z., Jiang Q., Zhou D., Yi J. (2024). Promoters, Key Cis-Regulatory Elements, and Their Potential Applications in Regulation of Cadmium (Cd) in Rice. Int. J. Mol. Sci..

[64] Romani F., Moreno J.E. (2021). Molecular Mechanisms Involved in Functional Macroevolution of Plant Transcription Factors. New Phytol..

[65] Zhang T., Lv W., Zhang H., Ma L., Li P., Ge L., Li G. (2018). Genome-Wide Analysis of the Basic Helix-Loop-Helix (bHLH) Transcription Factor Family in Maize. BMC Plant Biol..

[66] Ke Y.-Z., Wu Y.-W., Zhou H.-J., Chen P., Wang M.-M., Liu M.-M., Li P.-F., Yang J., Li J.-N., Du H. (2020). Genome-Wide Survey of the bHLH Super Gene Family in *Brassica napus*. BMC Plant Biol..

[67] Yanhui C., Xiaoyuan Y., Kun H., Meihua L., Jigang L., Zhaofeng G., Zhiqiang L., Yunfei Z., Xiaoxiao W., Xiaoming Q. (2006). The MYB Transcription Factor Superfamily of Arabidopsis: Expression Analysis and Phylogenetic Comparison with the Rice MYB Family. Plant Mol. Biol..

[68] Muthuramalingam P., Jeyasri R., Selvaraj A., Shin H., Chen J.-T., Satish L., Wu Q.-S., Ramesh M. (2022). Global Integrated Genomic and Transcriptomic Analyses of MYB Transcription Factor Superfamily in C3 Model Plant *Oryza sativa* (L.) Unravel Potential Candidates Involved in Abiotic Stress Signaling. Front. Genet..

[69] Kim M., Xi H., Park J. (2021). Genome-Wide Comparative Analyses of GATA Transcription Factors among 19 Arabidopsis Ecotype Genomes: Intraspecific Characteristics of GATA Transcription Factors. PLoS ONE.

[70] Peng W., Li W., Song N., Tang Z., Liu J., Wang Y., Pan S., Dai L., Wang B. (2021). Genome-Wide Characterization, Evolution, and Expression Profile Analysis of GATA Transcription Factors in *Brachypodium distachyon*. Int. J. Mol. Sci..

[71] Chen L., Yang Y., Liu C., Zheng Y., Xu M., Wu N., Sheng J., Shen L. (2015). Characterization of WRKY Transcription Factors in *Solanum lycopersicum* Reveals Collinearity and Their Expression Patterns under Cold Treatment. Biochem. Biophys. Res. Commun..

[72] Pan L.-J., Jiang L. (2014). Identification and Expression of the WRKY Transcription Factors of Carica Papaya in Response to Abiotic and Biotic Stresses. Mol. Biol. Rep..

[73] Dou L., Zhang X., Pang C., Song M., Wei H., Fan S., Yu S. (2014). Genome-Wide Analysis of the WRKY Gene Family in Cotton. Mol. Genet. Genom..

[74] Li W., Wan X.-L., Yu J.-Y., Wang K.-L., Zhang J. (2019). Genome-Wide Identification, Classification, and Expression Analysis of the Hsf Gene Family in Carnation (*Dianthus caryophyllus*). Int. J. Mol. Sci..

[75] Betts M.J., Guigó R., Agarwal P., Russell R.B. (2001). Exon Structure Conservation despite Low Sequence Similarity: A Relic of Dramatic Events in Evolution?. EMBO J..

[76] Marand A.P., Eveland A.L., Kaufmann K., Springer N.M. (2023). Cis-Regulatory Elements in Plant Development, Adaptation, and Evolution. Annu. Rev. Plant Biol..

[77] Park S.C., Kwon H.B., Shih M.C. (1996). Cis-Acting Elements Essential for Light Regulation of the Nuclear Gene Encoding the A Subunit of Chloroplast Glyceraldehyde 3-Phosphate Dehydrogenase in Arabidopsis Thaliana. Plant Physiol..

[78] Van de Peer Y., Ashman T.-L., Soltis P.S., Soltis D.E. (2021). Polyploidy: An Evolutionary and Ecological Force in Stressful Times. Plant Cell.

[79] Zou Y., Han C., Wang F., Tan Y., Yang S., Huang C., Xie S., Xiao X. (2021). Integrated Metabolome and Transcriptome Analysis Reveal Complex Molecular Mechanisms Underlying Selenium Response of *Aloe Vera* L.. J. Plant Biol..

[80] Guo L., Liao Y., Deng S., Li J., Bu X., Zhu C., Zhang W., Cong X., Cheng S., Chen Q. (2024). Genome-Wide Analysis of NAC Transcription Factors and Exploration of Candidate Genes Regulating Selenium Metabolism in *Broussonetia papyrifera*. Planta.

[81] Bian Y., Li X., Gao D., Zhang G., Zhang A., Feng Y., Hua Z., Liang L. (2025). Effects of Organic Selenium on Metabolic Responses and Disease Resistance in Rose Plants. J. Hazard. Mater..

