# Supplementary material for: Identification and Expression Analysis of Transcription Factor Family in Highland Barley Seedlings Under Na2SeO3 Treatment
Source: Life (Basel). 2026 Feb 2;16(2):255. doi: 10.3390/life16020255 (PMC12941567; doi:10.3390/life16020255)
Supplement: Supplementary file 1 [file life-16-00255-s001.zip › Figure S.pdf]

Figure A

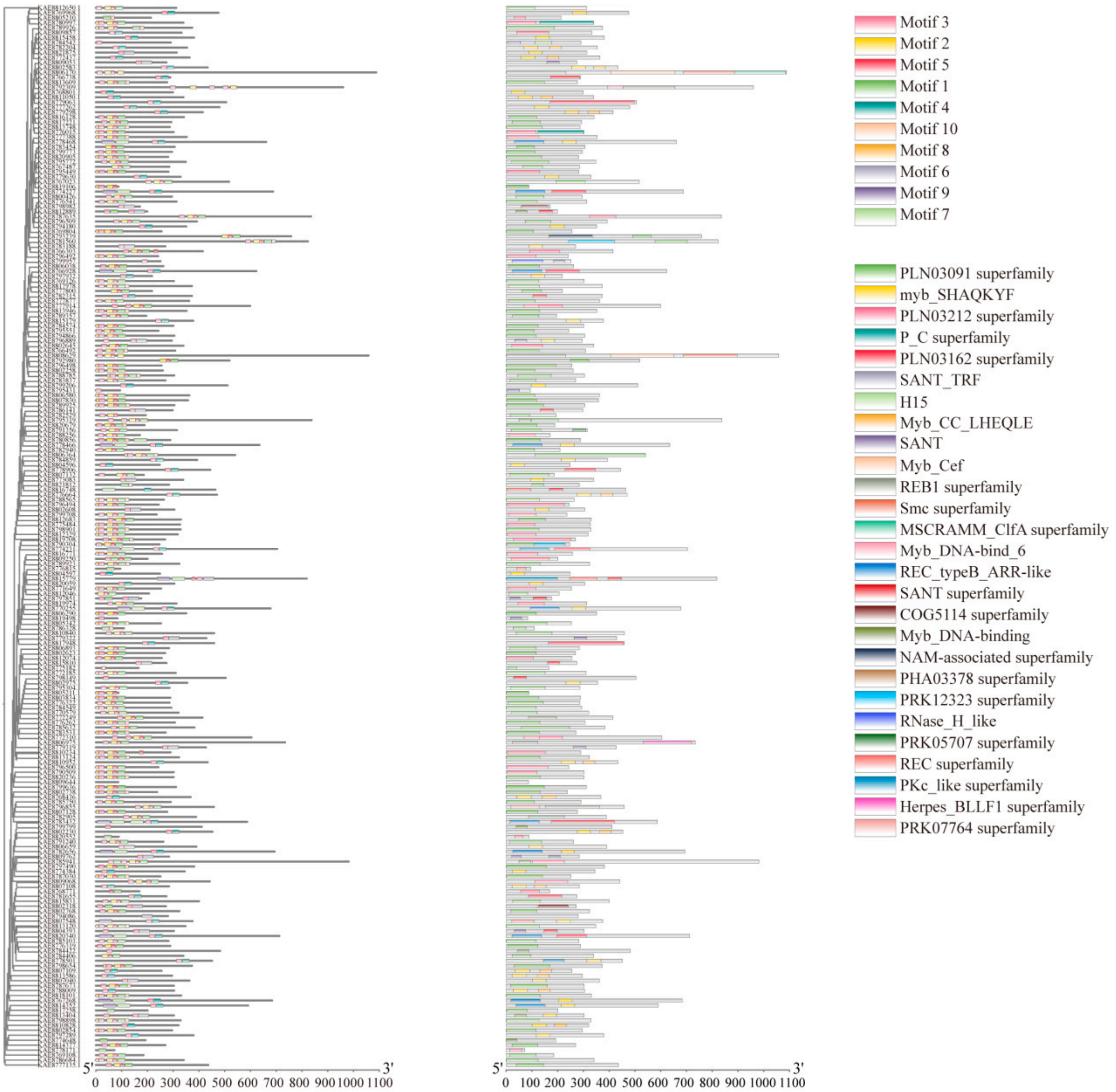
Motif 3
Motif 2
Motif 5
Motif 1
Motif 4
Motif 10
Motif 8
Motif 6
Motif 9
Motif 7
PLN03091 superfamily
myb_SHAQKYF
PLN03212 superfamily
P_C superfamily
PLN03162 superfamily
SANT_TRF
H15
Myb_CC_LHEQLE
SANT
Myb_Cef
REB1 superfamily
Smc superfamily
MSCRAMM_ClfA superfamily
Myb_DNA-bind_6
REC_typeB_ARR-like
SANT superfamily
COG5114 superfamily
Myb_DNA-binding
NAM-associated superfamily
PHA03378 superfamily
PRK12323 superfamily
RNase_H_like
PRK05707 superfamily
REC superfamily
PKc_like superfamily
Herpes_BLLF1 superfamily
PRK07764 superfamily
5'
3'
0 100 200 300 400 500 600 700 800 900 1000 1100
5'
3'
0 100 200 300 400 500 600 700 800 900 1000 1100

Figure B

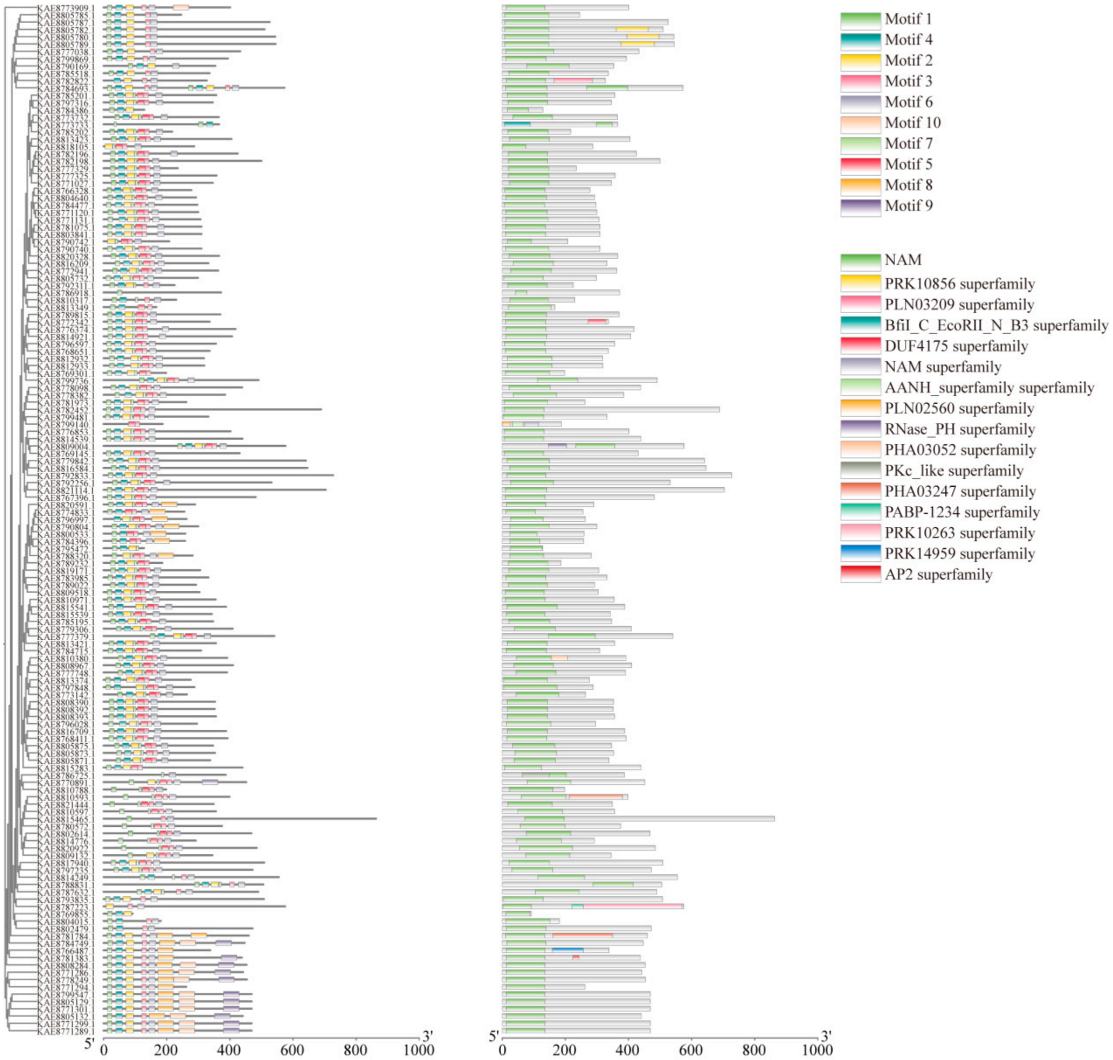

Figure S1 Phylogenetic tree, conserved motifs, and conserved domains of gene family in highland barley. (A) MYB. (B) NAC.

Figure A

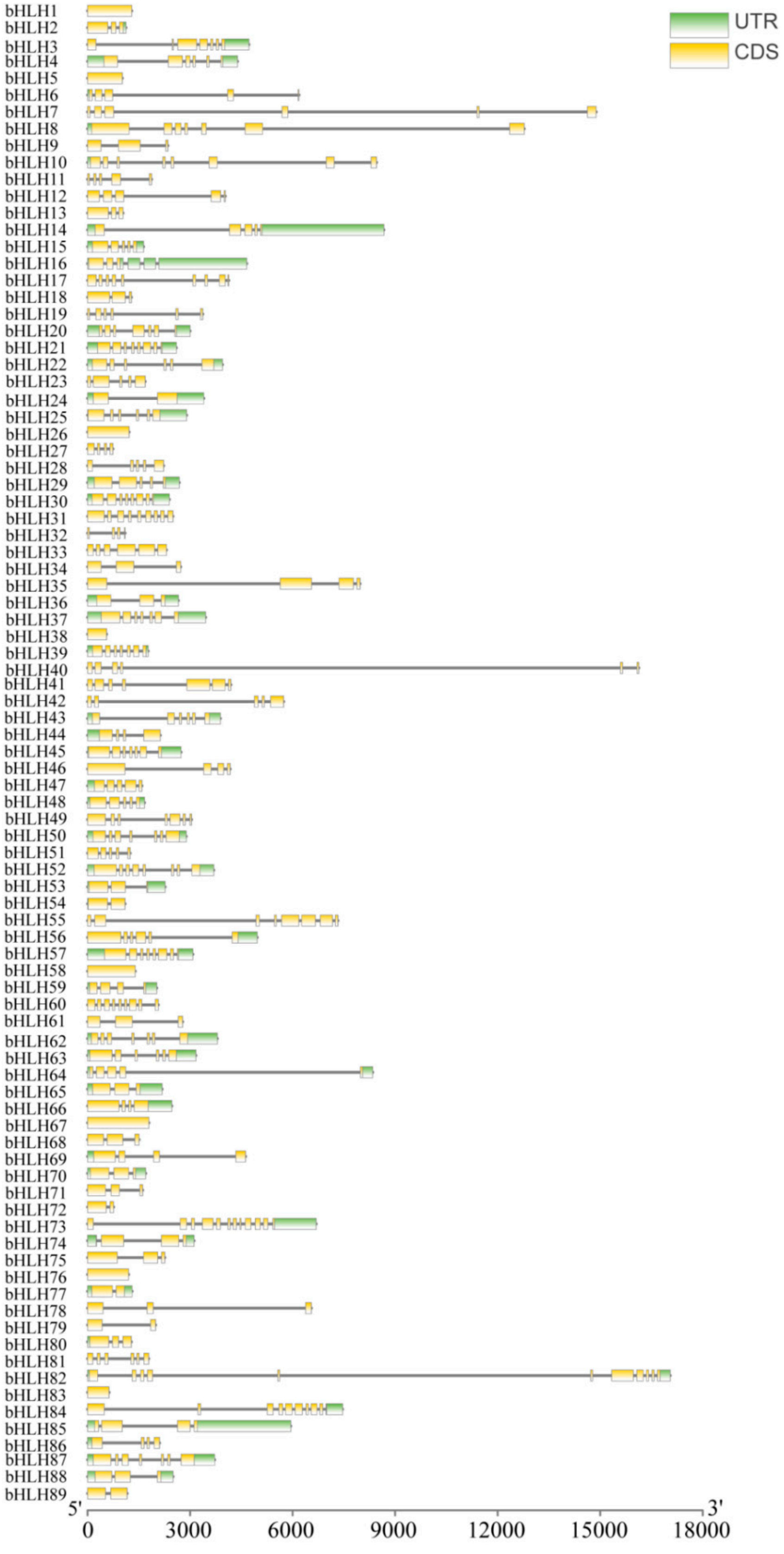
UTR
CDS
bHLH1
bHLH2
bHLH3
bHLH4
bHLH5
bHLH6
bHLH7
bHLH8
bHLH9
bHLH10
bHLH11
bHLH12
bHLH13
bHLH14
bHLH15
bHLH16
bHLH17
bHLH18
bHLH19
bHLH20
bHLH21
bHLH22
bHLH23
bHLH24
bHLH25
bHLH26
bHLH27
bHLH28
bHLH29
bHLH30
bHLH31
bHLH32
bHLH33
bHLH34
bHLH35
bHLH36
bHLH37
bHLH38
bHLH39
bHLH40
bHLH41
bHLH42
bHLH43
bHLH44
bHLH45
bHLH46
bHLH47
bHLH48
bHLH49
bHLH50
bHLH51
bHLH52
bHLH53
bHLH54
bHLH55
bHLH56
bHLH57
bHLH58
bHLH59
bHLH60
bHLH61
bHLH62
bHLH63
bHLH64
bHLH65
bHLH66
bHLH67
bHLH68
bHLH69
bHLH70
bHLH71
bHLH72
bHLH73
bHLH74
bHLH75
bHLH76
bHLH77
bHLH78
bHLH79
bHLH80
bHLH81
bHLH82
bHLH83
bHLH84
bHLH85
bHLH86
bHLH87
bHLH88
bHLH89
5'
3'
0
3000
6000
9000
12000
15000
18000

Figure B

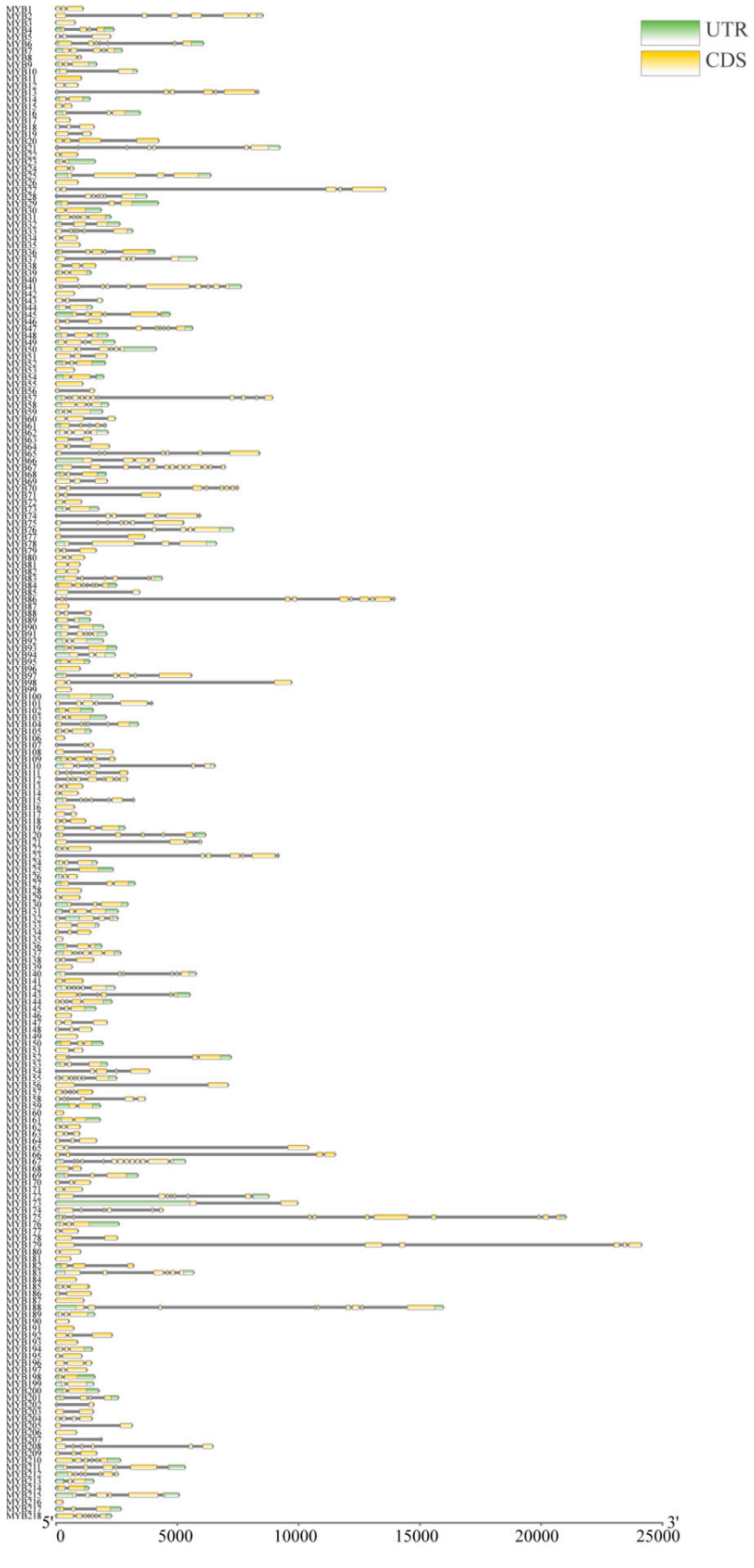

Figure C

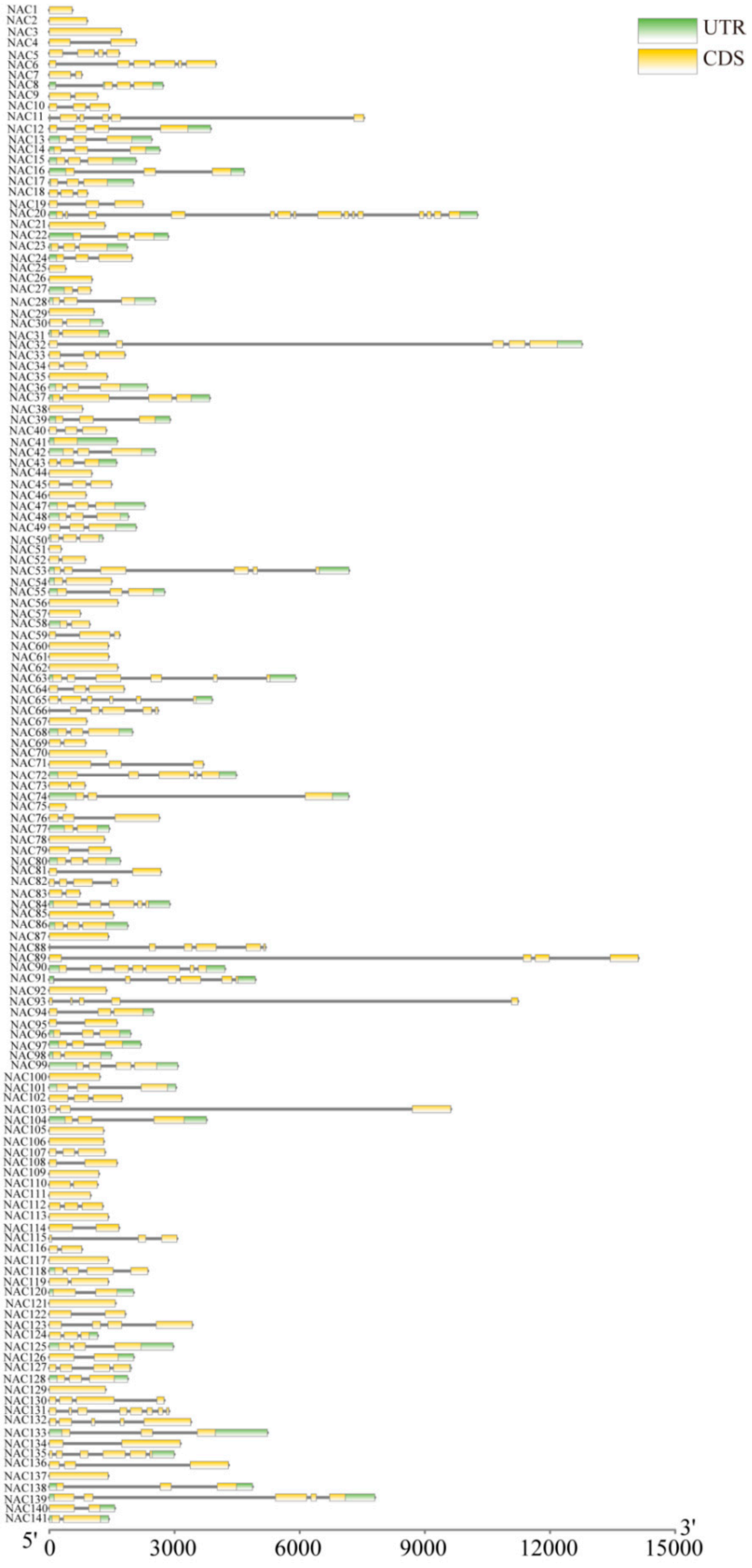

Figure D

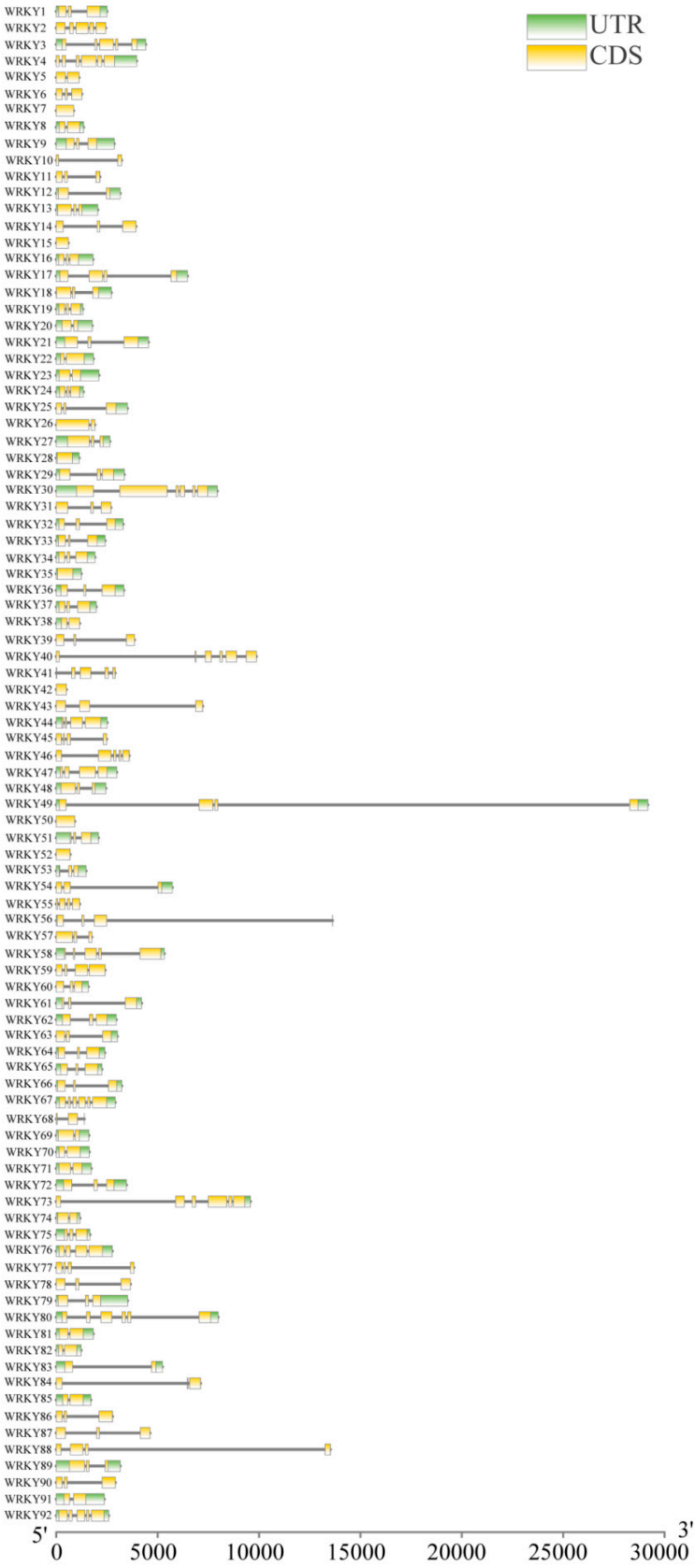
UTR
CDS
WRKY1
WRKY2
WRKY3
WRKY4
WRKY5
WRKY6
WRKY7
WRKY8
WRKY9
WRKY10
WRKY11
WRKY12
WRKY13
WRKY14
WRKY15
WRKY16
WRKY17
WRKY18
WRKY19
WRKY20
WRKY21
WRKY22
WRKY23
WRKY24
WRKY25
WRKY26
WRKY27
WRKY28
WRKY29
WRKY30
WRKY31
WRKY32
WRKY33
WRKY34
WRKY35
WRKY36
WRKY37
WRKY38
WRKY39
WRKY40
WRKY41
WRKY42
WRKY43
WRKY44
WRKY45
WRKY46
WRKY47
WRKY48
WRKY49
WRKY50
WRKY51
WRKY52
WRKY53
WRKY54
WRKY55
WRKY56
WRKY57
WRKY58
WRKY59
WRKY60
WRKY61
WRKY62
WRKY63
WRKY64
WRKY65
WRKY66
WRKY67
WRKY68
WRKY69
WRKY70
WRKY71
WRKY72
WRKY73
WRKY74
WRKY75
WRKY76
WRKY77
WRKY78
WRKY79
WRKY80
WRKY81
WRKY82
WRKY83
WRKY84
WRKY85
WRKY86
WRKY87
WRKY88
WRKY89
WRKY90
WRKY91
WRKY92
5'
3'
0
5000
10000
15000
20000
25000
30000

Figure E

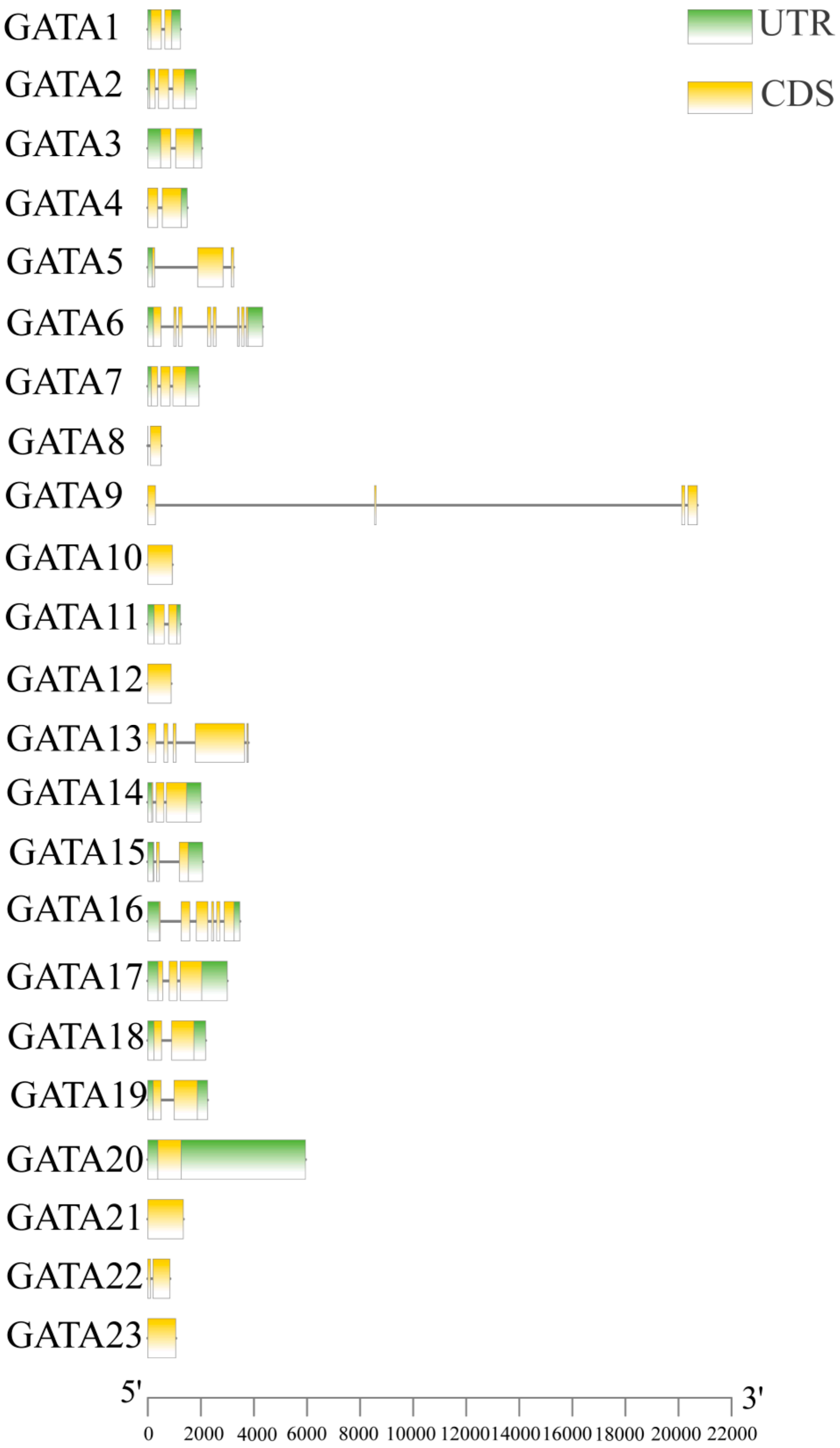
GATA1
GATA2
GATA3
GATA4
GATA5
GATA6
GATA7
GATA8
GATA9
GATA10
GATA11
GATA12
GATA13
GATA14
GATA15
GATA16
GATA17
GATA18
GATA19
GATA20
GATA21
GATA22
GATA23
UTR
CDS
5'
3'
0 2000 4000 6000 8000 10000 12000 14000 16000 18000 20000 22000

Figure F

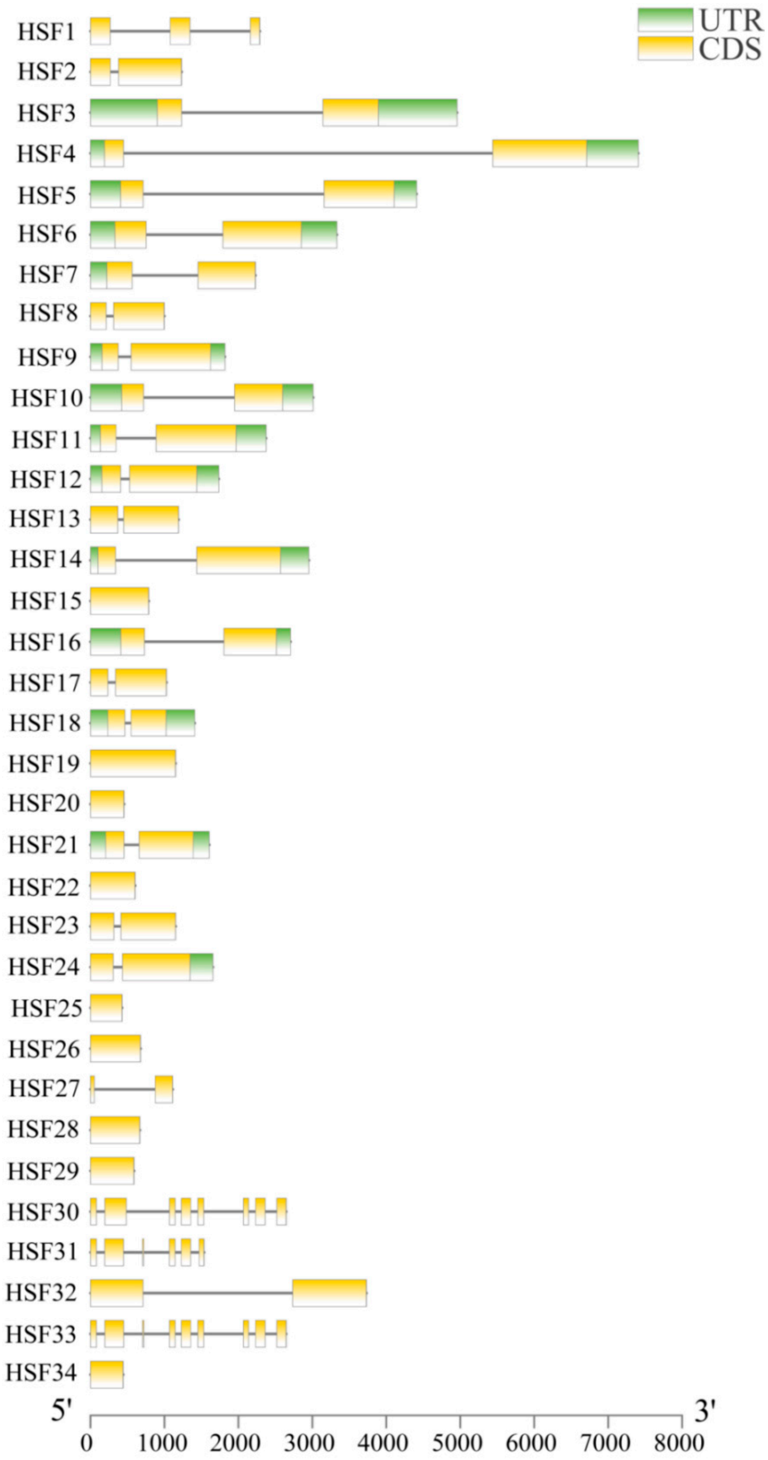

Figure S2 Gene structure of highland barley transcription factor family members. (A) bHLH. (B) MYB. (C) NAC. (D) WRKY. (E) GATA. (F) HSF.

Figure A

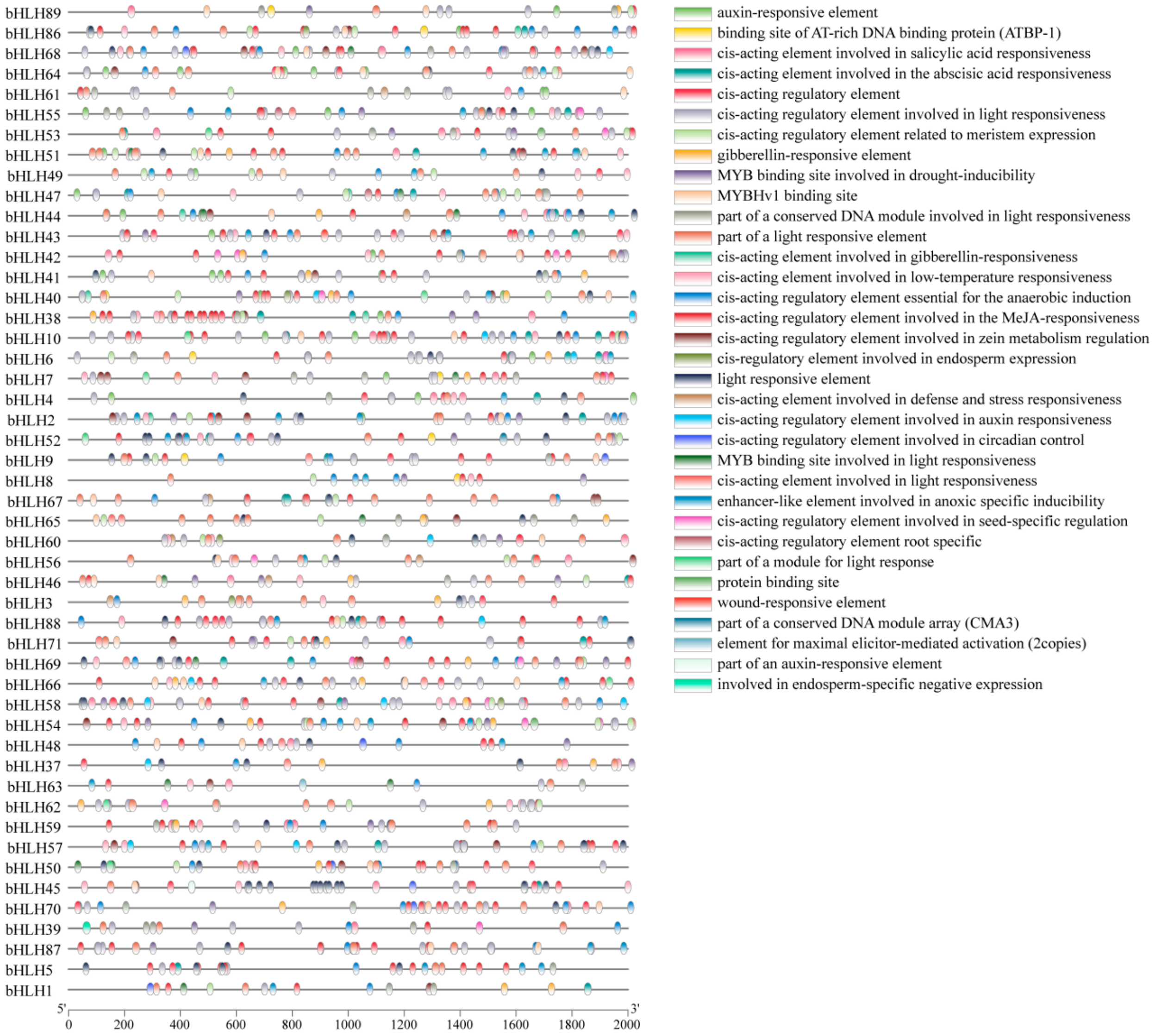
bHLH89
bHLH86
bHLH68
bHLH64
bHLH61
bHLH55
bHLH53
bHLH51
bHLH49
bHLH47
bHLH44
bHLH43
bHLH42
bHLH41
bHLH40
bHLH38
bHLH10
bHLH6
bHLH7
bHLH4
bHLH2
bHLH52
bHLH9
bHLH8
bHLH67
bHLH65
bHLH60
bHLH56
bHLH46
bHLH3
bHLH88
bHLH71
bHLH69
bHLH66
bHLH58
bHLH54
bHLH48
bHLH37
bHLH63
bHLH62
bHLH59
bHLH57
bHLH50
bHLH45
bHLH70
bHLH39
bHLH87
bHLH5
bHLH1
5'
3'
0
200
400
600
800
1000
1200
1400
1600
1800
2000
auxin-responsive element
binding site of AT-rich DNA binding protein (ATBP-1)
cis-acting element involved in salicylic acid responsiveness
cis-acting element involved in the abscisic acid responsiveness
cis-acting regulatory element
cis-acting regulatory element involved in light responsiveness
cis-acting regulatory element related to meristem expression
gibberellin-responsive element
MYB binding site involved in drought-inducibility
MYBHv1 binding site
part of a conserved DNA module involved in light responsiveness
part of a light responsive element
cis-acting element involved in gibberellin-responsiveness
cis-acting element involved in low-temperature responsiveness
cis-acting regulatory element essential for the anaerobic induction
cis-acting regulatory element involved in the MeJA-responsiveness
cis-acting regulatory element involved in zein metabolism regulation
cis-regulatory element involved in endosperm expression
light responsive element
cis-acting element involved in defense and stress responsiveness
cis-acting regulatory element involved in auxin responsiveness
cis-acting regulatory element involved in circadian control
MYB binding site involved in light responsiveness
cis-acting element involved in light responsiveness
enhancer-like element involved in anoxic specific inducibility
cis-acting regulatory element involved in seed-specific regulation
cis-acting regulatory element root specific
part of a module for light response
protein binding site
wound-responsive element
part of a conserved DNA module array (CMA3)
element for maximal elicitor-mediated activation (2copies)
part of an auxin-responsive element
involved in endosperm-specific negative expression

Figure B

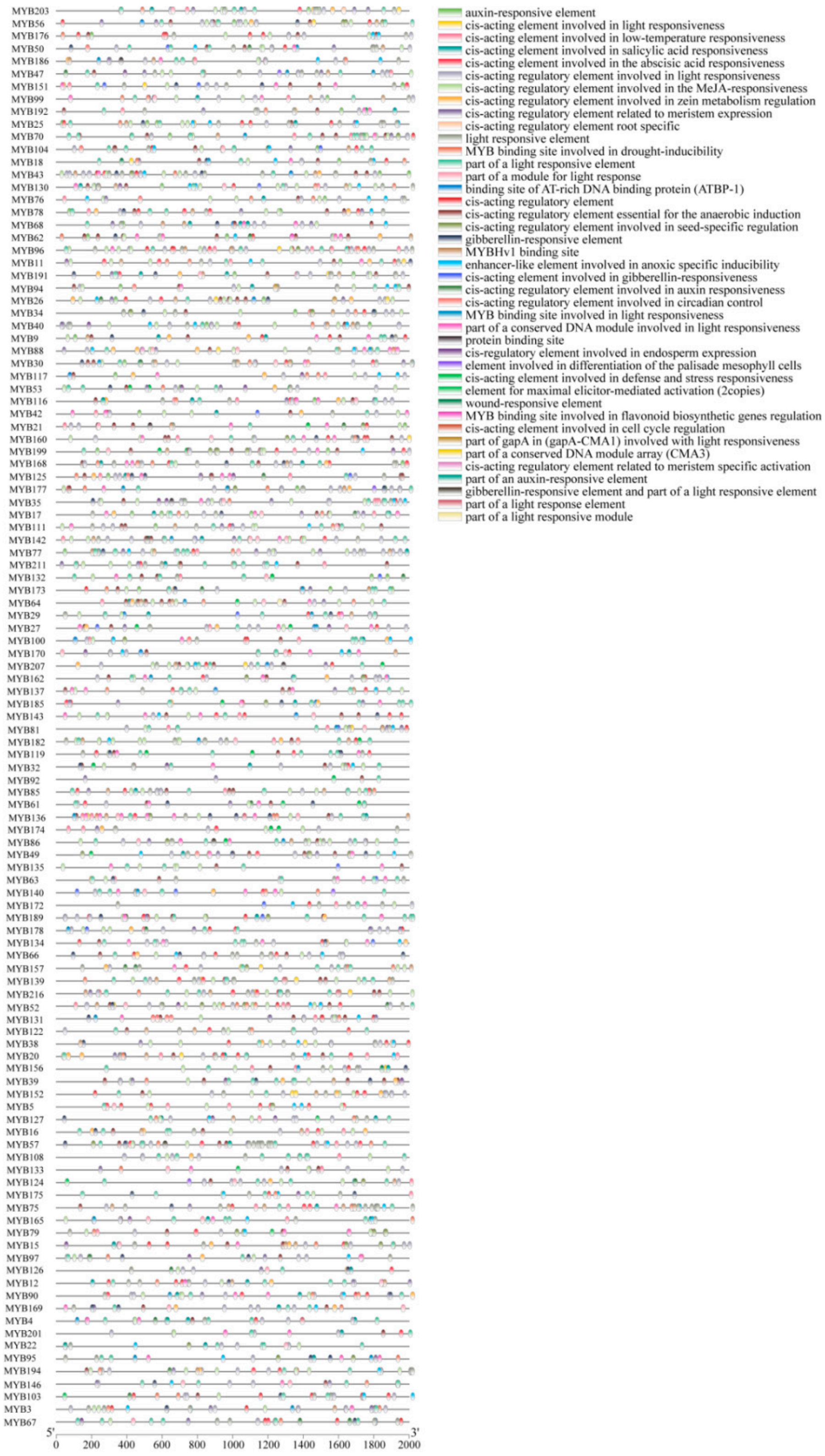
MYB203
MYB56
MYB176
MYB50
MYB186
MYB47
MYB151
MYB99
MYB192
MYB25
MYB70
MYB104
MYB18
MYB43
MYB130
MYB76
MYB78
MYB68
MYB62
MYB96
MYB11
MYB191
MYB94
MYB26
MYB34
MYB40
MYB9
MYB88
MYB30
MYB117
MYB53
MYB116
MYB42
MYB21
MYB160
MYB199
MYB168
MYB125
MYB177
MYB35
MYB17
MYB111
MYB142
MYB77
MYB211
MYB132
MYB173
MYB64
MYB29
MYB27
MYB100
MYB170
MYB207
MYB162
MYB137
MYB185
MYB143
MYB81
MYB182
MYB119
MYB32
MYB92
MYB85
MYB61
MYB136
MYB174
MYB86
MYB49
MYB135
MYB63
MYB140
MYB172
MYB189
MYB178
MYB134
MYB66
MYB157
MYB139
MYB216
MYB52
MYB131
MYB122
MYB38
MYB20
MYB156
MYB39
MYB152
MYB5
MYB127
MYB16
MYB57
MYB108
MYB133
MYB124
MYB175
MYB75
MYB165
MYB79
MYB15
MYB97
MYB126
MYB12
MYB90
MYB169
MYB4
MYB201
MYB22
MYB95
MYB194
MYB146
MYB103
MYB3
MYB67
5'
3'
0
200
400
600
800
1000
1200
1400
1600
1800
2000
auxin-responsive element
cis-acting element involved in light responsiveness
cis-acting element involved in low-temperature responsiveness
cis-acting element involved in salicylic acid responsiveness
cis-acting element involved in the abscisic acid responsiveness
cis-acting regulatory element involved in light responsiveness
cis-acting regulatory element involved in the MeJA-responsiveness
cis-acting regulatory element involved in zein metabolism regulation
cis-acting regulatory element related to meristem expression
cis-acting regulatory element root specific
light responsive element
MYB binding site involved in drought-inducibility
part of a light responsive element
part of a module for light response
binding site of AT-rich DNA binding protein (ATBP-1)
cis-acting regulatory element
cis-acting regulatory element essential for the anaerobic induction
cis-acting regulatory element involved in seed-specific regulation
gibberellin-responsive element
MYBHv1 binding site
enhancer-like element involved in anoxic specific inducibility
cis-acting element involved in gibberellin-responsiveness
cis-acting regulatory element involved in auxin responsiveness
cis-acting regulatory element involved in circadian control
MYB binding site involved in light responsiveness
part of a conserved DNA module involved in light responsiveness
protein binding site
cis-regulatory element involved in endosperm expression
element involved in differentiation of the palisade mesophyll cells
cis-acting element involved in defense and stress responsiveness
element for maximal elicitor-mediated activation (2copies)
wound-responsive element
MYB binding site involved in flavonoid biosynthetic genes regulation
cis-acting element involved in cell cycle regulation
part of gapA in (gapA-CMA1) involved with light responsiveness
part of a conserved DNA module array (CMA3)
cis-acting regulatory element related to meristem specific activation
part of an auxin-responsive element
gibberellin-responsive element and part of a light responsive element
part of a light response element
part of a light responsive module

Figure C

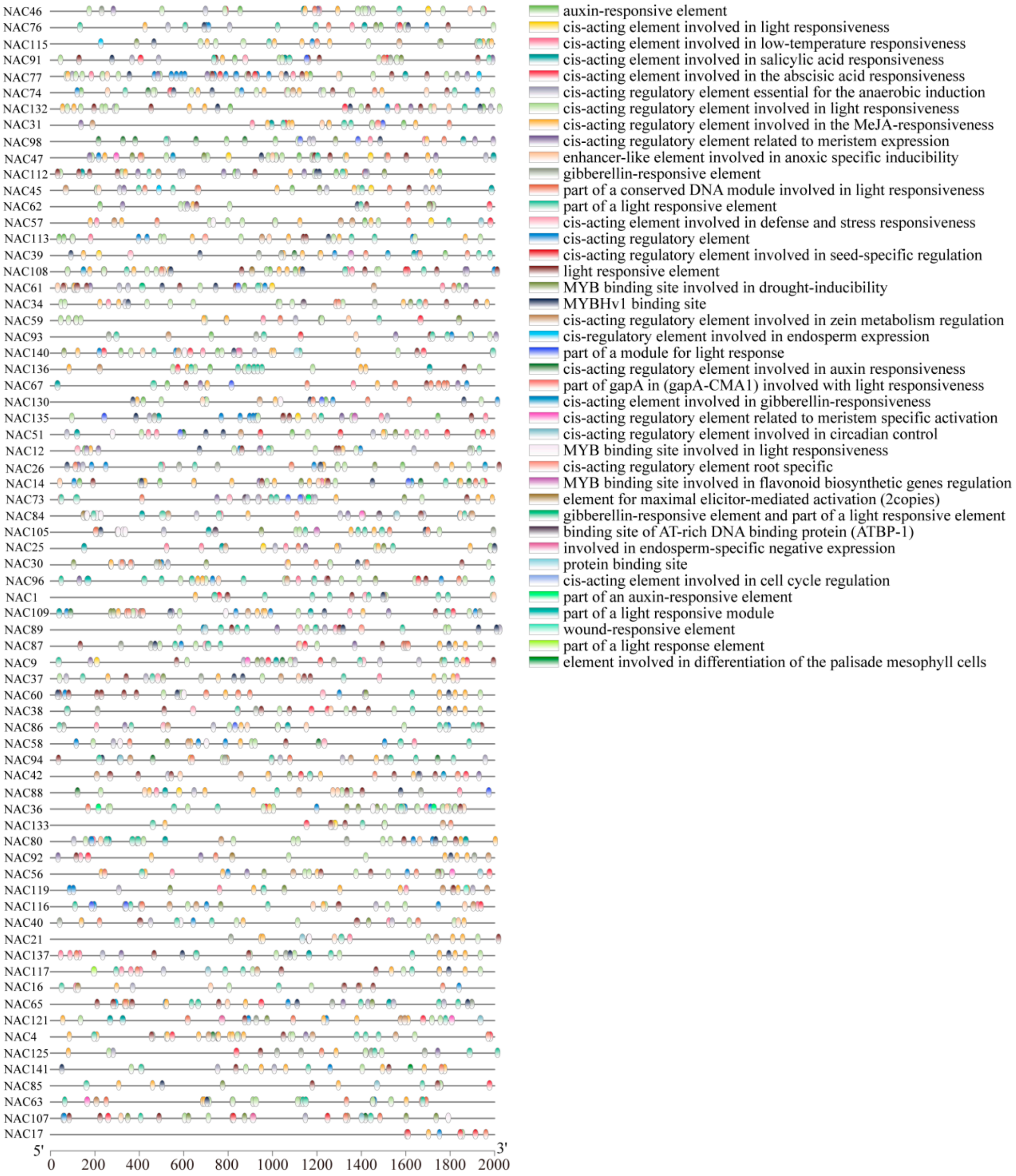
NAC46
NAC76
NAC115
NAC91
NAC77
NAC74
NAC132
NAC31
NAC98
NAC47
NAC112
NAC45
NAC62
NAC57
NAC113
NAC39
NAC108
NAC61
NAC34
NAC59
NAC93
NAC140
NAC136
NAC67
NAC130
NAC135
NAC51
NAC12
NAC26
NAC14
NAC73
NAC84
NAC105
NAC25
NAC30
NAC96
NAC1
NAC109
NAC89
NAC87
NAC9
NAC37
NAC60
NAC38
NAC86
NAC58
NAC94
NAC42
NAC88
NAC36
NAC133
NAC80
NAC92
NAC56
NAC119
NAC116
NAC40
NAC21
NAC137
NAC117
NAC16
NAC65
NAC121
NAC4
NAC125
NAC141
NAC85
NAC63
NAC107
NAC17
5'
3'
0
200
400
600
800
1000
1200
1400
1600
1800
2000
auxin-responsive element
cis-acting element involved in light responsiveness
cis-acting element involved in low-temperature responsiveness
cis-acting element involved in salicylic acid responsiveness
cis-acting element involved in the abscisic acid responsiveness
cis-acting regulatory element essential for the anaerobic induction
cis-acting regulatory element involved in light responsiveness
cis-acting regulatory element involved in the MeJA-responsiveness
cis-acting regulatory element related to meristem expression
enhancer-like element involved in anoxic specific inducibility
gibberellin-responsive element
part of a conserved DNA module involved in light responsiveness
part of a light responsive element
cis-acting element involved in defense and stress responsiveness
cis-acting regulatory element
cis-acting regulatory element involved in seed-specific regulation
light responsive element
MYB binding site involved in drought-inducibility
MYBHv1 binding site
cis-acting regulatory element involved in zein metabolism regulation
cis-regulatory element involved in endosperm expression
part of a module for light response
cis-acting regulatory element involved in auxin responsiveness
part of gapA in (gapA-CMA1) involved with light responsiveness
cis-acting element involved in gibberellin-responsiveness
cis-acting regulatory element related to meristem specific activation
cis-acting regulatory element involved in circadian control
MYB binding site involved in light responsiveness
cis-acting regulatory element root specific
MYB binding site involved in flavonoid biosynthetic genes regulation
element for maximal elicitor-mediated activation (2copies)
gibberellin-responsive element and part of a light responsive element
binding site of AT-rich DNA binding protein (ATBP-1)
involved in endosperm-specific negative expression
protein binding site
cis-acting element involved in cell cycle regulation
part of an auxin-responsive element
part of a light responsive module
wound-responsive element
part of a light response element
element involved in differentiation of the palisade mesophyll cells

Figure D

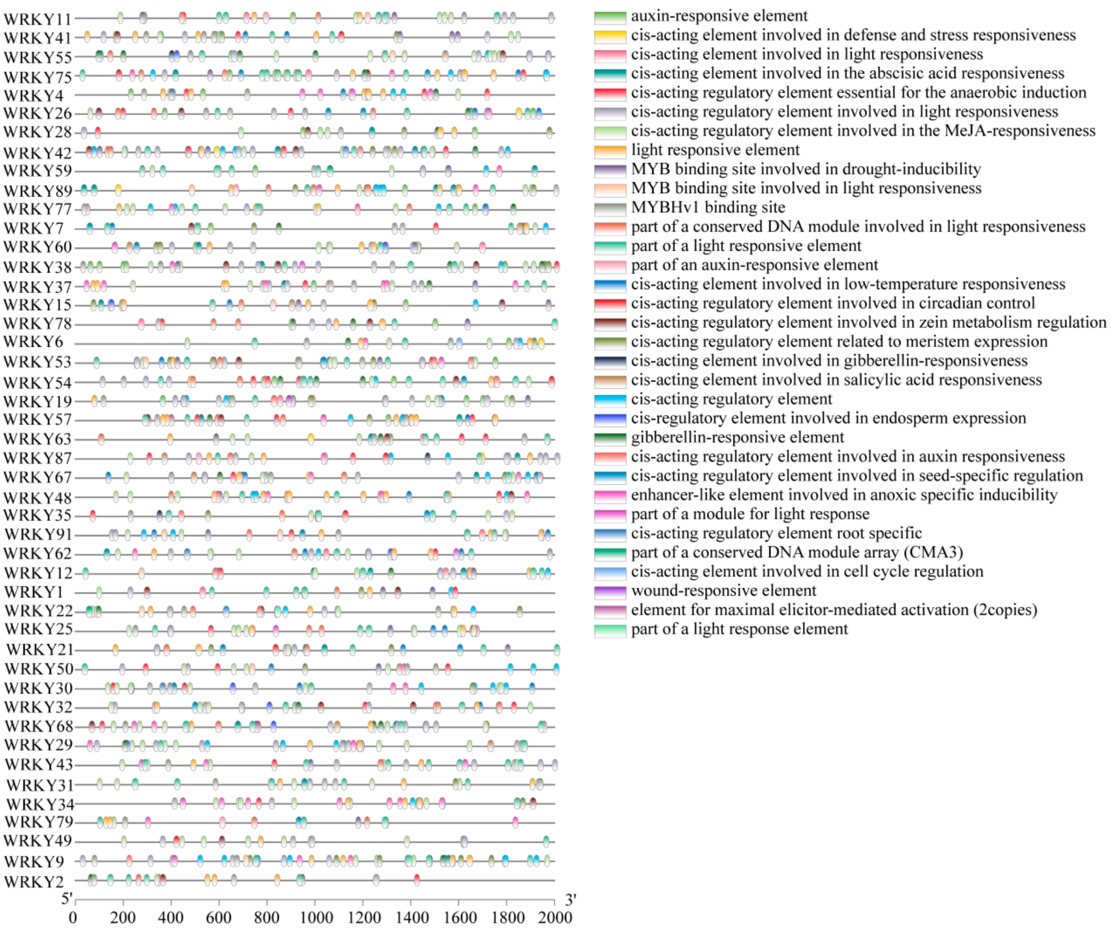
WRKY11
WRKY41
WRKY55
WRKY75
WRKY4
WRKY26
WRKY28
WRKY42
WRKY59
WRKY89
WRKY77
WRKY7
WRKY60
WRKY38
WRKY37
WRKY15
WRKY78
WRKY6
WRKY53
WRKY54
WRKY19
WRKY57
WRKY63
WRKY87
WRKY67
WRKY48
WRKY35
WRKY91
WRKY62
WRKY12
WRKY1
WRKY22
WRKY25
WRKY21
WRKY50
WRKY30
WRKY32
WRKY68
WRKY29
WRKY43
WRKY31
WRKY34
WRKY79
WRKY49
WRKY9
WRKY2
5'
3'
0
200
400
600
800
1000
1200
1400
1600
1800
2000
auxin-responsive element
cis-acting element involved in defense and stress responsiveness
cis-acting element involved in light responsiveness
cis-acting element involved in the abscisic acid responsiveness
cis-acting regulatory element essential for the anaerobic induction
cis-acting regulatory element involved in light responsiveness
cis-acting regulatory element involved in the MeJA-responsiveness
light responsive element
MYB binding site involved in drought-inducibility
MYB binding site involved in light responsiveness
MYBHv1 binding site
part of a conserved DNA module involved in light responsiveness
part of a light responsive element
part of an auxin-responsive element
cis-acting element involved in low-temperature responsiveness
cis-acting regulatory element involved in circadian control
cis-acting regulatory element involved in zein metabolism regulation
cis-acting regulatory element related to meristem expression
cis-acting element involved in gibberellin-responsiveness
cis-acting element involved in salicylic acid responsiveness
cis-acting regulatory element
cis-regulatory element involved in endosperm expression
gibberellin-responsive element
cis-acting regulatory element involved in auxin responsiveness
cis-acting regulatory element involved in seed-specific regulation
enhancer-like element involved in anoxic specific inducibility
part of a module for light response
cis-acting regulatory element root specific
part of a conserved DNA module array (CMA3)
cis-acting element involved in cell cycle regulation
wound-responsive element
element for maximal elicitor-mediated activation (2copies)
part of a light response element

Figure E

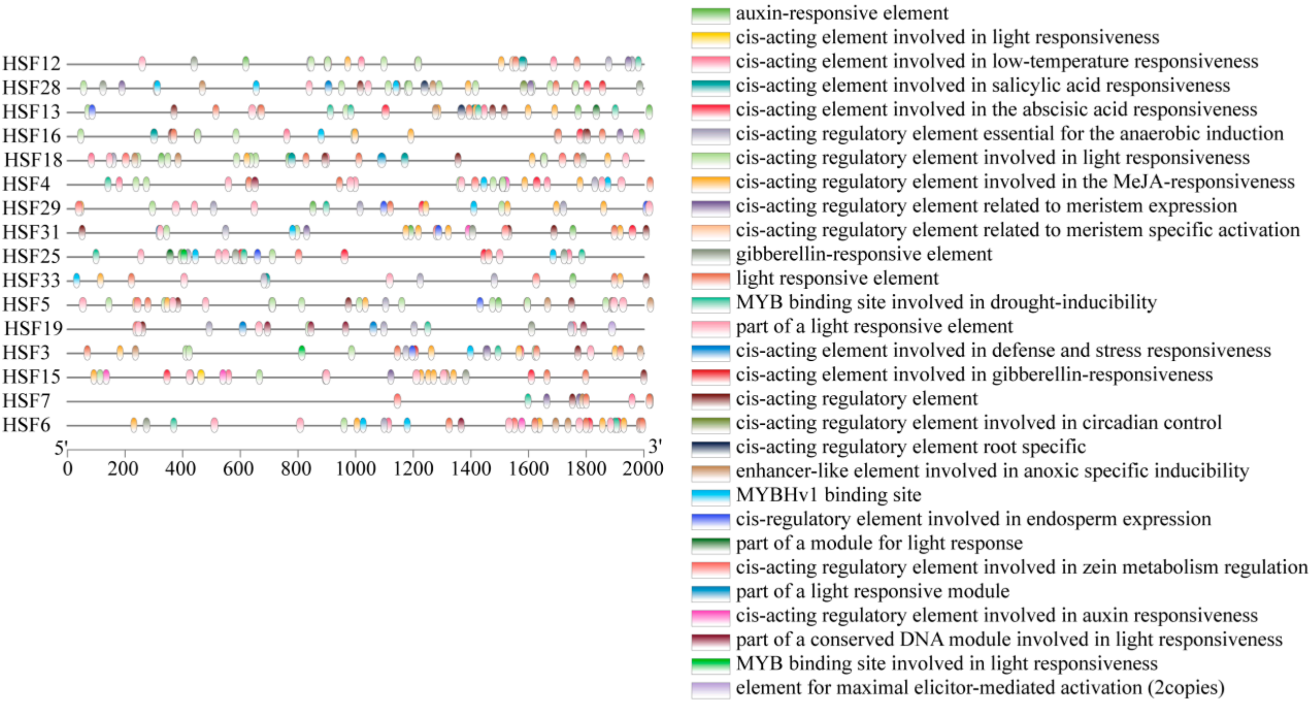

HSF12
HSF28
HSF13
HSF16
HSF18
HSF4
HSF29
HSF31
HSF25
HSF33
HSF5
HSF19
HSF3
HSF15
HSF7
HSF6
5'
3'
0
200
400
600
800
1000
1200
1400
1600
1800
2000
auxin-responsive element
cis-acting element involved in light responsiveness
cis-acting element involved in low-temperature responsiveness
cis-acting element involved in salicylic acid responsiveness
cis-acting element involved in the abscisic acid responsiveness
cis-acting regulatory element essential for the anaerobic induction
cis-acting regulatory element involved in light responsiveness
cis-acting regulatory element involved in the MeJA-responsiveness
cis-acting regulatory element related to meristem expression
cis-acting regulatory element related to meristem specific activation
gibberellin-responsive element
light responsive element
MYB binding site involved in drought-inducibility
part of a light responsive element
cis-acting element involved in defense and stress responsiveness
cis-acting element involved in gibberellin-responsiveness
cis-acting regulatory element
cis-acting regulatory element involved in circadian control
cis-acting regulatory element root specific
enhancer-like element involved in anoxic specific inducibility
MYBHv1 binding site
cis-regulatory element involved in endosperm expression
part of a module for light response
cis-acting regulatory element involved in zein metabolism regulation
part of a light responsive module
cis-acting regulatory element involved in auxin responsiveness
part of a conserved DNA module involved in light responsiveness
MYB binding site involved in light responsiveness
element for maximal elicitor-mediated activation (2copies)

Figure F

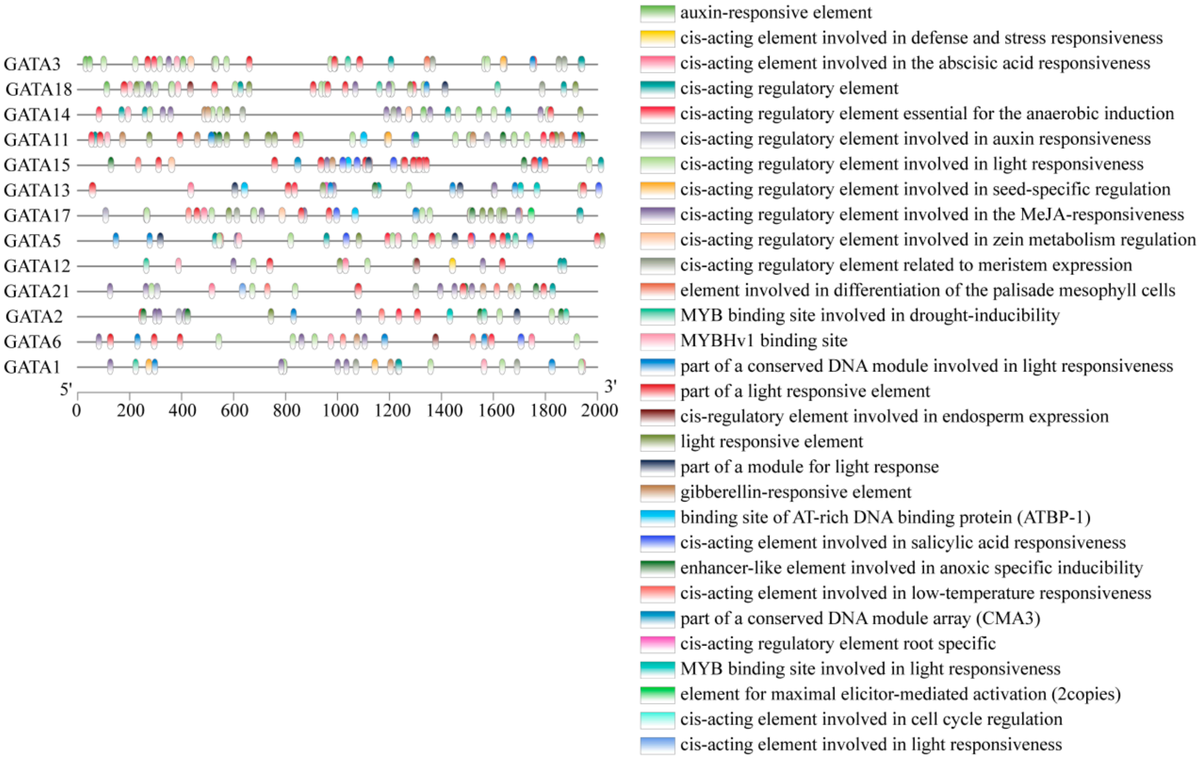

Figure S3 Analysis of cis-acting elements of highland barley transcription factor family members. (A) bHLH. (B) MYB. (C) NAC. (D) WRKY. (E) GATA. (F) HSF.

Figure A

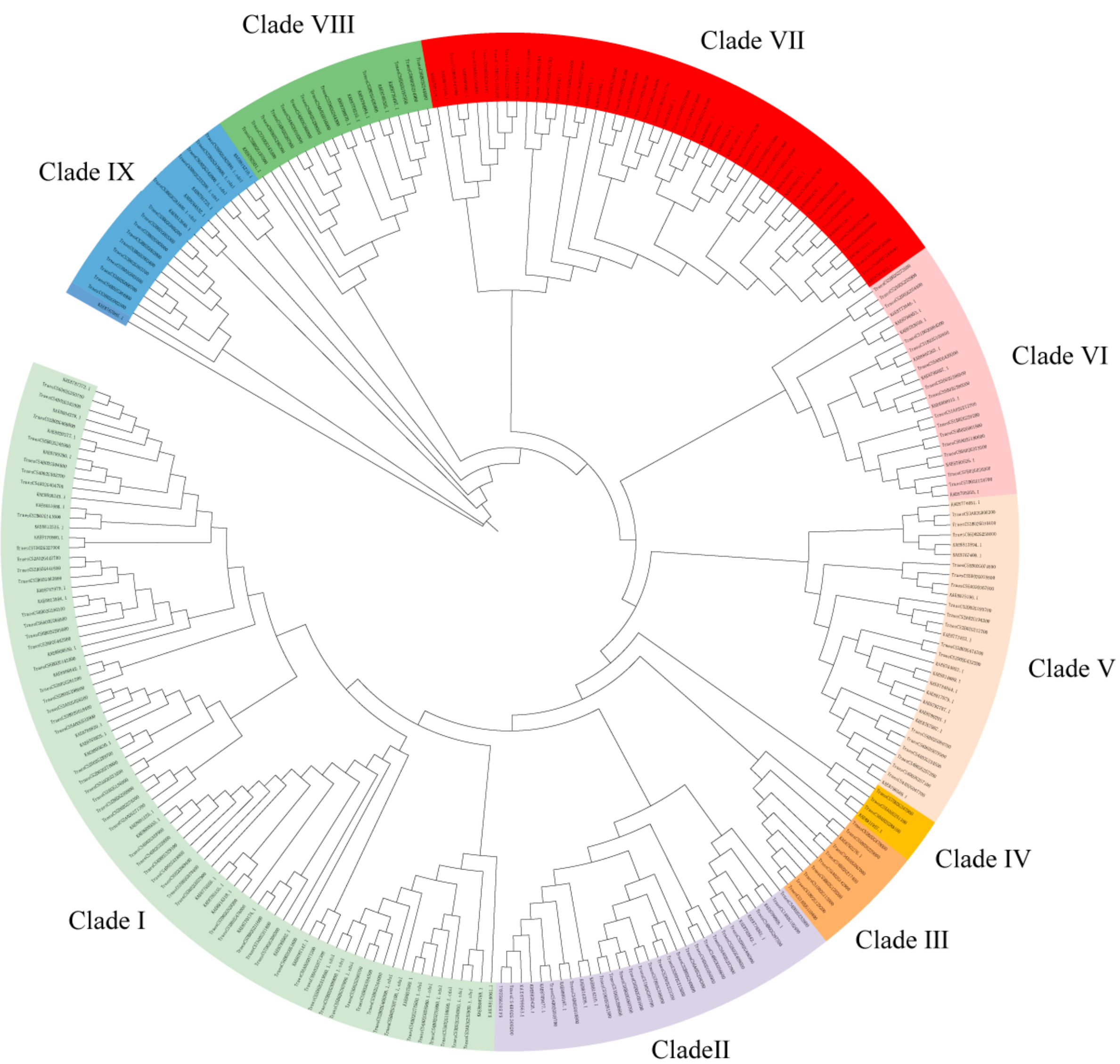

Clade VIII
Clade VII
Clade IX
Clade VI
Clade V
Clade IV
Clade III
CladeII
Clade I

Figure B

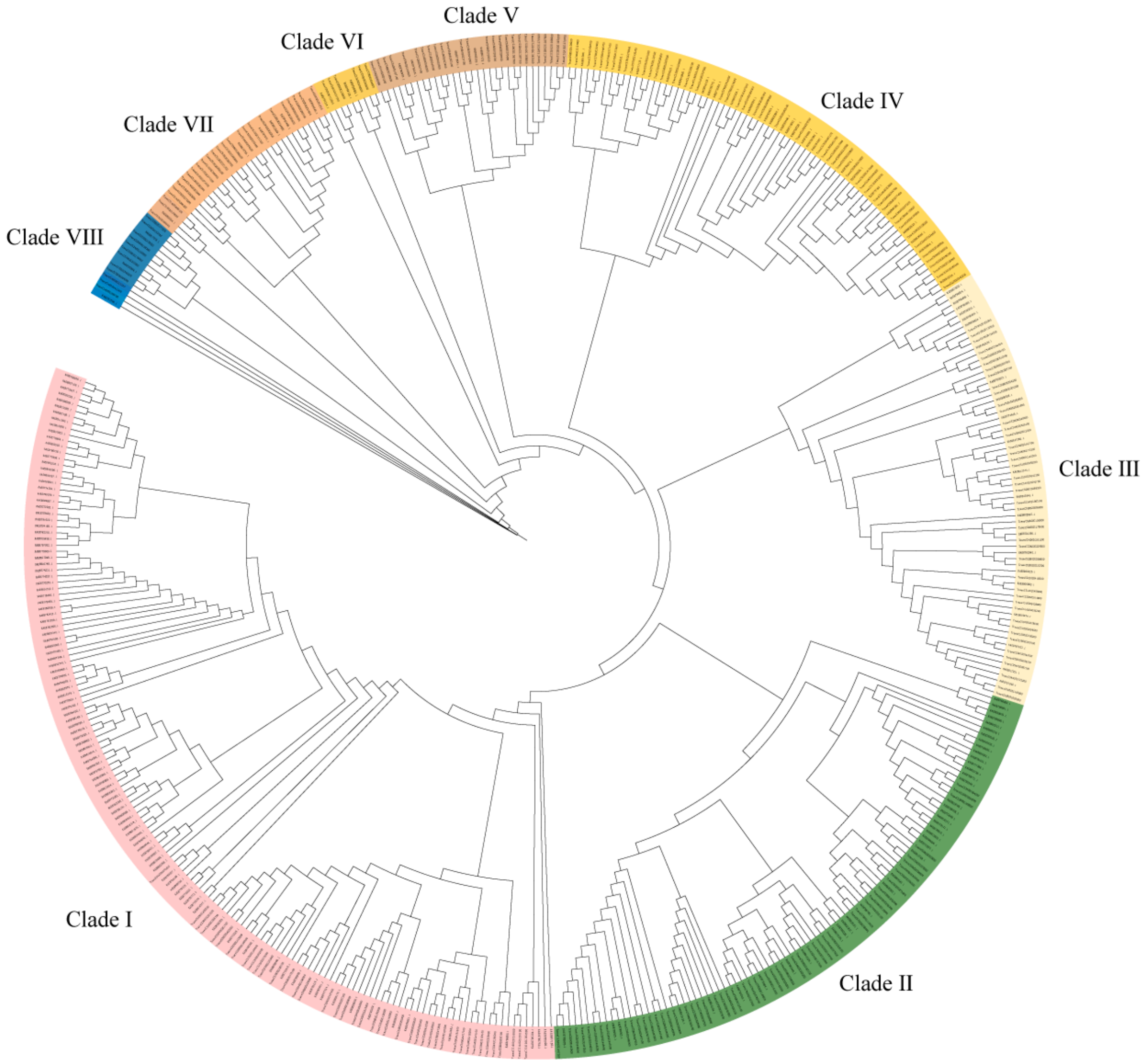
Clade V
Clade VI
Clade VII
Clade VIII
Clade IV
Clade III
Clade I
Clade II

Figure C

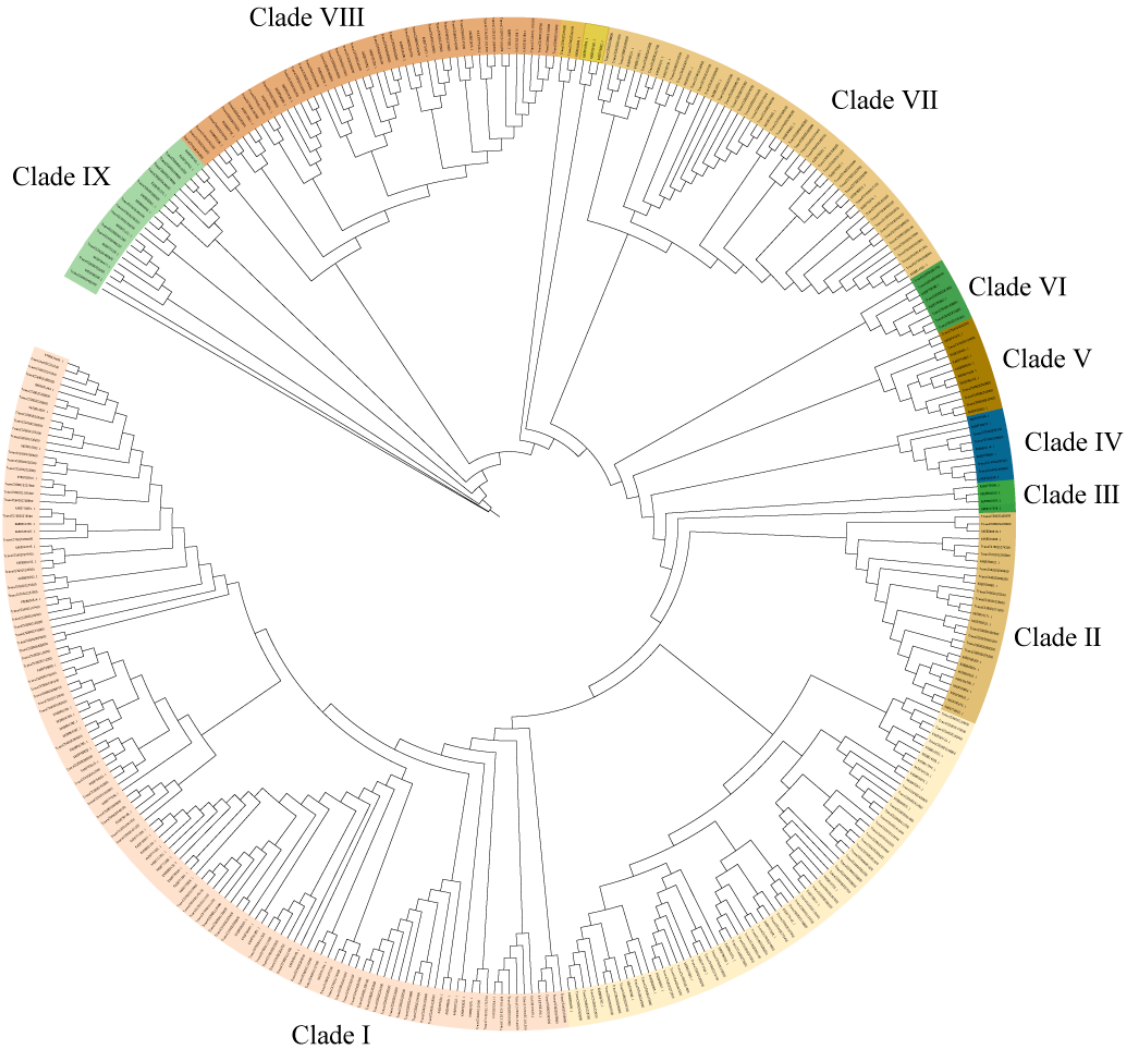
Clade VIII
Clade VII
Clade IX
Clade VI
Clade V
Clade IV
Clade III
Clade II
Clade I

Figure D

Clade IV

Clade III

Clade V

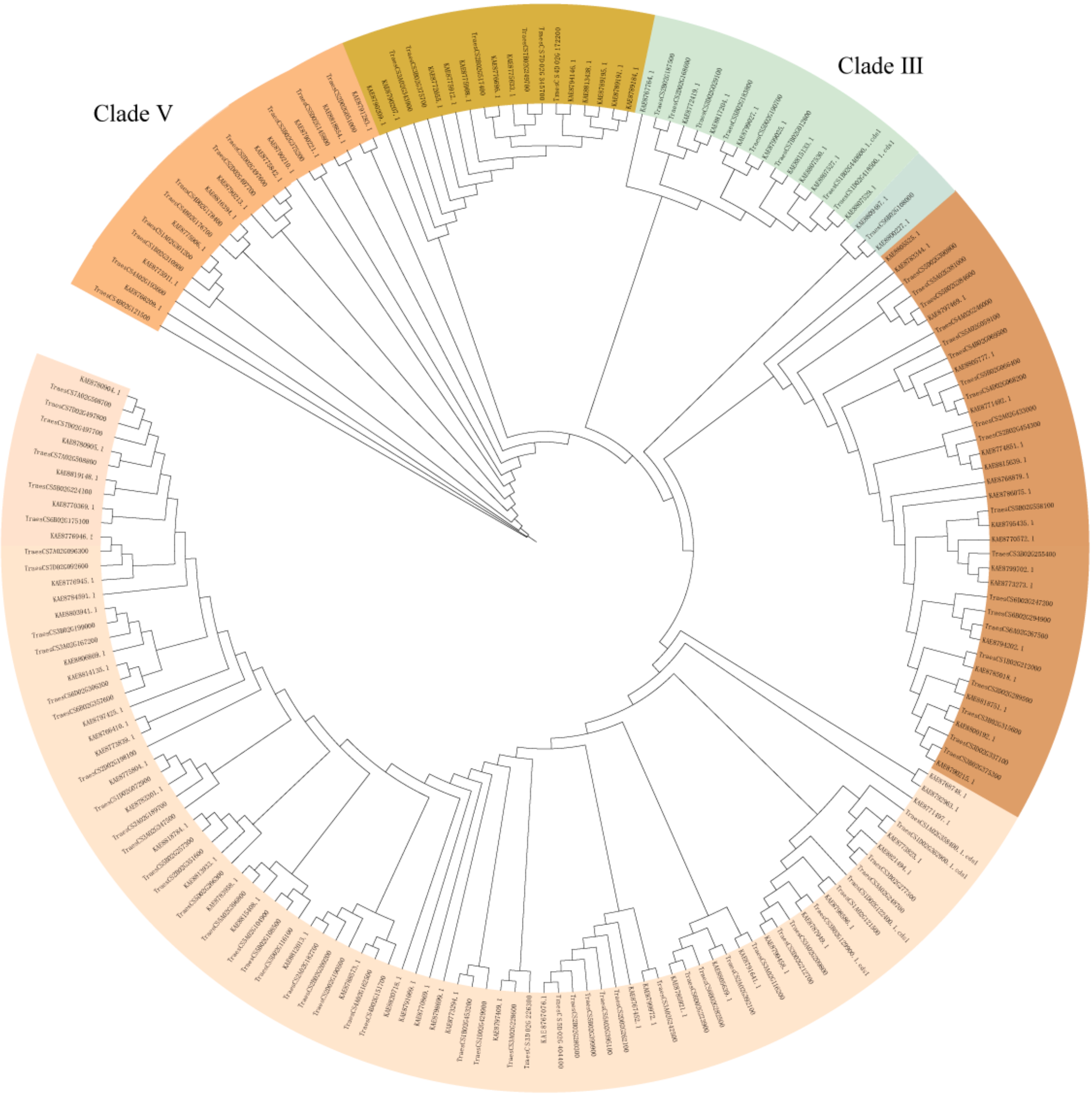

Clade II

Clade I

Figure E

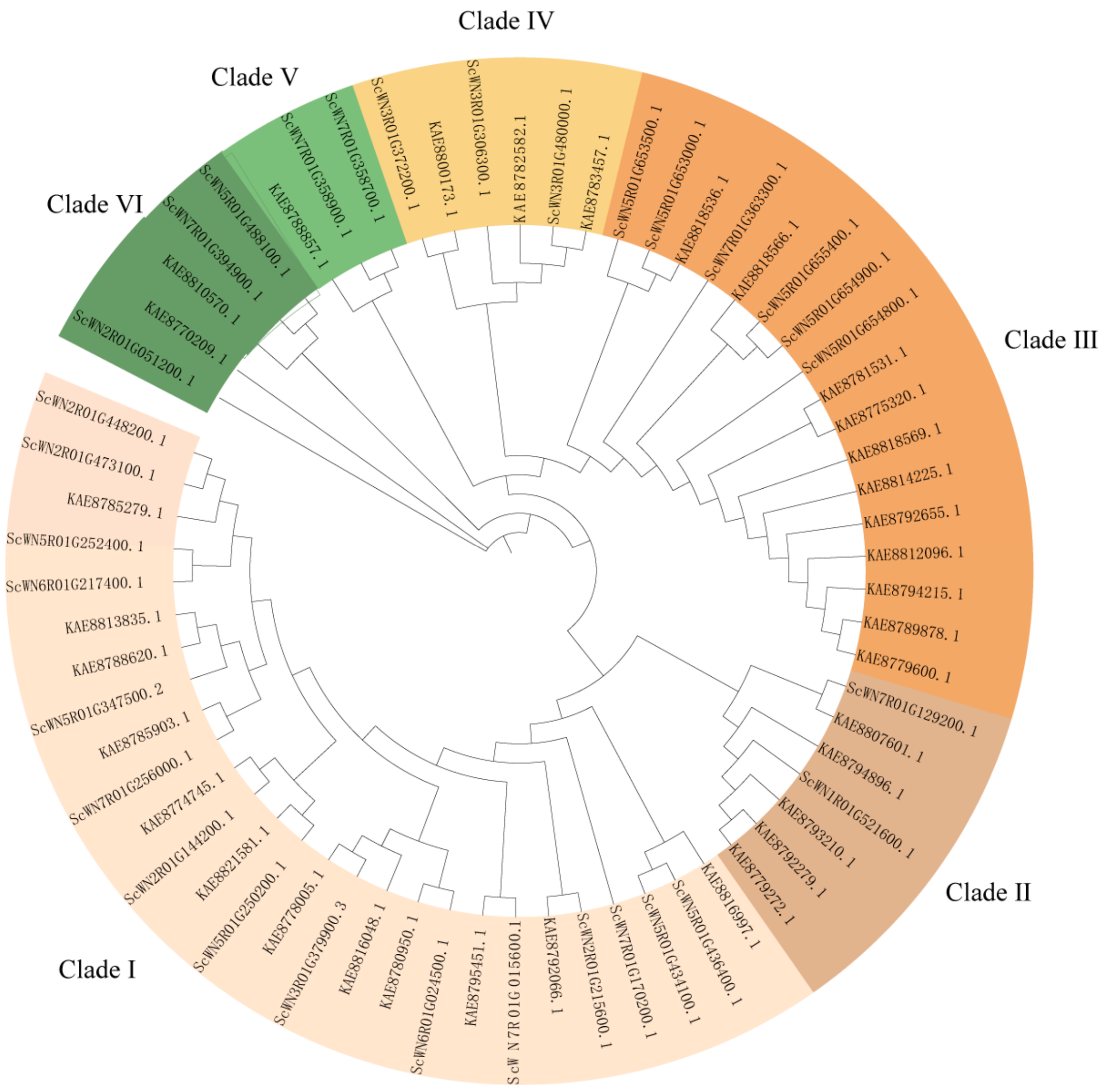
Clade IV
Clade V
Clade VI
Clade III
Clade II
Clade I
ScWN2R01G051200. 1
KAE8770209. 1
KAE8810570. 1
ScWN7R01G394900. 1
ScWN5R01G488100. 1
KAE8788857. 1
ScWN7R01G358900. 1
ScWN7R01G358700. 1
ScWN3R01G372200. 1
KAE8800173. 1
ScWN3R01G306300. 1
KAE8782582.1
ScWN3R01G480000. 1
KAE8783457. 1
ScWN5R01G653500. 1
ScWN5R01G653000. 1
KAE8818536. 1
ScWN7R01G363300. 1
KAE8818566. 1
ScWN5R01G655400. 1
ScWN5R01G654900. 1
ScWN5R01G654800. 1
KAE8781531. 1
KAE8775320. 1
KAE8818569. 1
KAE8814225. 1
KAE8792655. 1
KAE8812096. 1
KAE8794215. 1
KAE8789878. 1
KAE8779600. 1
ScWN7R01G129200. 1
KAE8807601. 1
KAE8794896. 1
ScWN1R01G521600. 1
KAE8793210. 1
KAE8792279. 1
KAE8779272. 1
KAE8816997. 1
ScWN5R01G436400. 1
ScWN5R01G434100. 1
ScWN7R01G170200. 1
ScWN2R01G215600. 1
KAE8792066. 1
ScW N7R01G 015600.1
KAE8795451. 1
ScWN6R01G024500. 1
KAE8780950. 1
KAE8816048. 1
ScWN3R01G379900. 3
KAE8778005. 1
ScWN5R01G250200. 1
KAE8821581. 1
ScWN2R01G144200. 1
KAE8774745. 1
ScWN7R01G256000. 1
KAE8785903. 1
ScWN5R01G347500. 2
KAE8788620. 1
KAE8813835. 1
ScWN6R01G217400. 1
ScWN5R01G252400. 1
KAE8785279. 1
ScWN2R01G473100. 1
ScWN2R01G448200. 1

Figure F

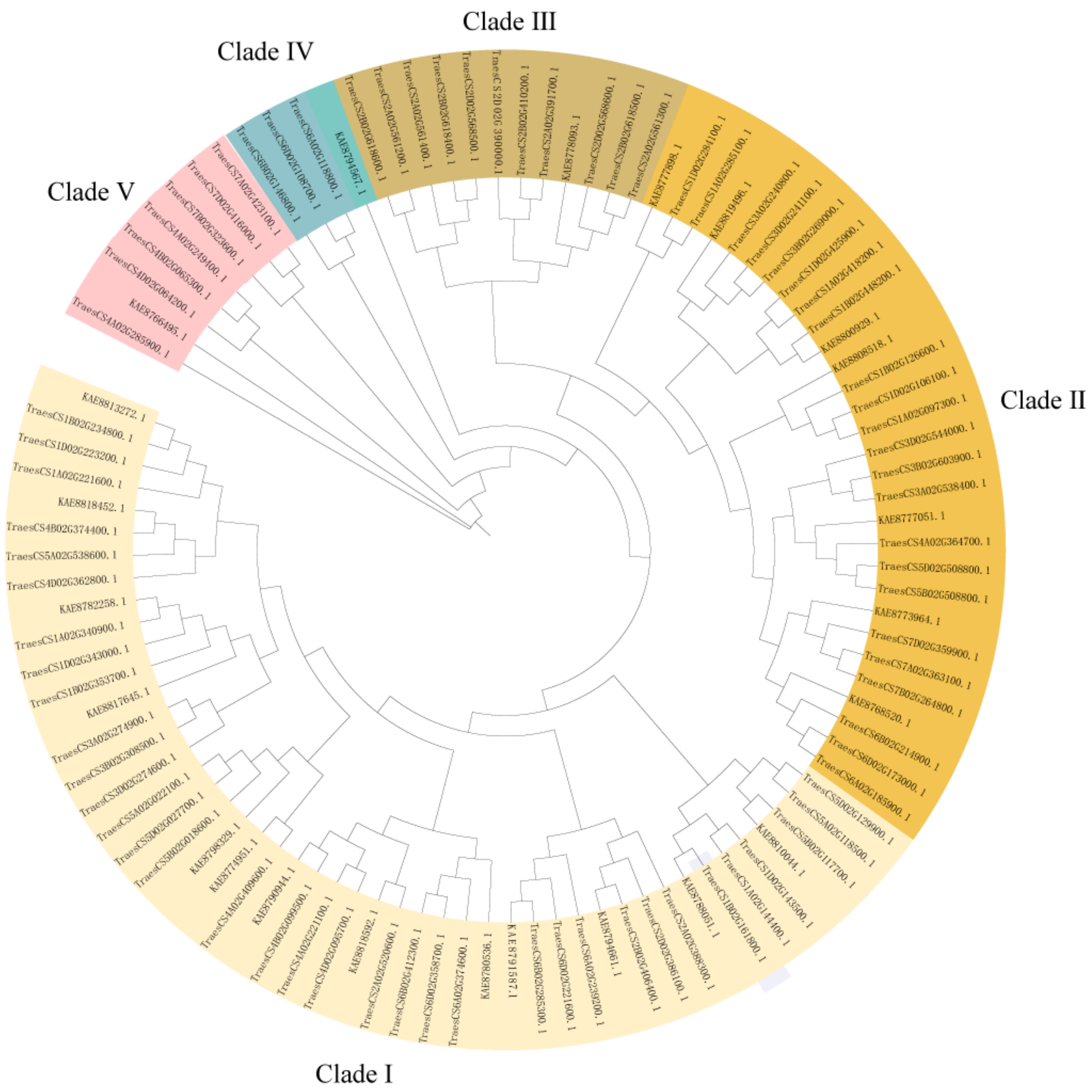

Figure S4 Phylogenetic tree of transcription factor genes in highland barley and wheat. (A) bHLH. (B) MYB. (C) NAC. (D) WRKY. (E) GATA. (F) HSF. Used IQ tree to construct the evolutionary tree using the maximum likelihood method (bootstrap value: 1000 repetitions).
